# Molecular organization of the distal tip of vertebrate motile cilia

**DOI:** 10.1101/2025.02.19.639145

**Published:** 2025-02-19

**Authors:** Juyeon Hong, Chanjae Lee, Ophelia Papoulas, Jiehong Pan, Maki Takagishi, Nadia Manzi, Daniel J. Dickinson, Amjad Horani, Steven L. Brody, Edward Marcotte, Tae Joo Park, John B. Wallingford

**Affiliations:** 1. Department of Molecular Biosciences, University of Texas at Austin, Texas, USA; 2. Department of Biological Sciences, Ulsan National Institute of Science and Technology, Ulsan, South Korea; 3. Division of Pulmonary and Critical Care Medicine, Department of Medicine, Washington University School of Medicine in St. Louis, USA; 4. Dept. of Medicinal and Life Sciences, Nagoya City University, Nagoya, Japan; 5. Division of Allergy and Pulmonary Medicine, Department of Pediatrics, Washington University School of Medicine in St. Louis, USA; 6. Center for Genomic Integrity, Institute for Basic Science, Ulsan 44919, Republic of Korea

## Abstract

The beating of cilia on multi-ciliated cells (MCCs) is essential for normal development and homeostasis in animals. Unlike basal bodies or axonemes, the distal tips of MCC cilia remain poorly defined. Here, we characterize the molecular organization of the distal tip of vertebrate MCC cilia, revealing two distinct domains occupied by distinct protein constituents. Using frog, mouse, and human MCCs, we find that two largely uncharacterized proteins, Ccdc78 and Ccdc33 occupy a specialized region at the extreme distal tip, and these are required for the normal organization of other tip proteins, including Spef1, Cep104, and Eb3. Ccdc78 and Cccdc33 are also independently required for normal length regulation of MCC cilia. Mechanistically, Ccdc78 and Ccdc33 display robust microtubule-bundling activity both *in vivo* and *in vitro*. Thus, we reveal that two previously undefined proteins form a key module for organizing and stabilizing the distal tip of motile cilia in vertebrate MCC. We propose that these proteins represent potential disease loci for motile ciliopathies.

## Introduction

**Figure 1.**
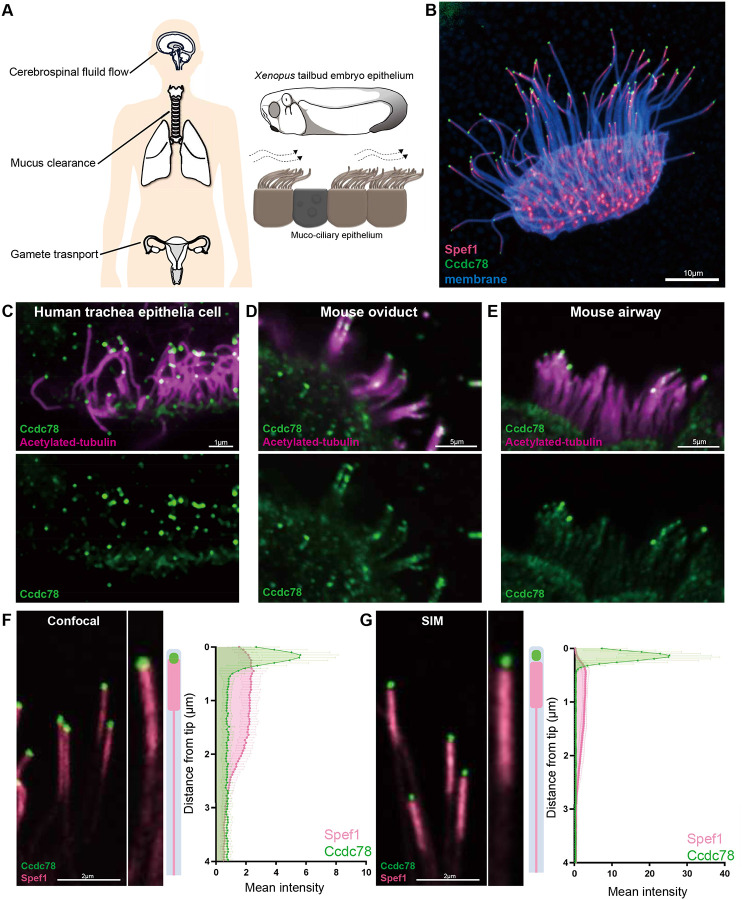
Ccdc78 localizes to the tip of motile cilia in MCCs (A) Schematic representations of the role of MCCs in the human brain, respiratory tract and reproductive system. The tailbud stage of *Xenopus* embryo is covered by mucociliary epithelium, which resembles mammalian MCCs. (B) Image of *Xenopus* MCC labeled with Spef1-RFP (magenta), GFP-Ccdc78 (green) and membrane-BFP (blue). Scale bar represents 10μm. (C) Immunofluorescence image of human trachea epithelial cells stained with anti-acetylated tubulin (magenta) and anti-Ccdc78 (green). Scale bar represents 1μm. (D-E) Imaging of mouse oviduct MCCs (D) and airway MCCs (E) stained with anti-acetylated tubulin (magenta) and anti-Ccdc78 (green). Scale bars represent 5μm. (F) Confocal image of cilia labeled with GFP-Ccdc78 (green) and Spef1-RFP (magenta), magnified view of the cilium on the right with cartoon. Quantification of fluorescent intensity along the axoneme shown in right, the mean intensity was normalized by average intensity (n=40 cilia). We quantified its localization in *Xenopus* by measuring signal intensity in the distal-most four microns of individual cilia, which allowed us to ignore confounding signals as crowded cilia lay cross one another more proximally on MCCs. (G) Structural illumination microscopy (SIM) image of cilia labeled with GFP-Ccdc78 (green) and Spef1-RFP (magenta), magnified view of the cilium on the right with cartoon. Quantification of fluorescent intensity along the axoneme shown in right (see Methods)(n=50 cilia).

Motile cilia are microtubule-based cellular organelles conserved across the eukaryotes and play important roles in development and homeostasis. Motile cilia on multi-ciliated cells (MCCs) generate fluid flow to clear the mucus in the airways, to transport the gametes in both male and female reproductive tracts, and to move cerebrospinal fluid in the central nervous system (Fig. 1A)^1^. Dysfunction of ciliary motility in MCCs causes primary ciliary dyskinesia (PCD), an incurable genetic disease characterized by infertility, hydrocephalus, and chronic respiratory tract infection, leading to bronchiectasis^2–4^.

Motile cilia in MCCs are unique for their myriad well-described specializations, from the rootlets and basal feet anchoring basal bodies to the carefully patterned positions of dynein motors in the axoneme. Far less is known, however, about the specializations at the distal tips of MCC cilia. This dearth of knowledge stems in part from the fact that these structures are far more variable than are other elements of motile cilia. Indeed, tip structures vary between species, between different tissues, and even between developmental stages of an organism^5^. In general, the normal microtubule pattern (9+2) is disorganized at the distal end, with the microtubule doublets each becoming singlets, and the central pair extending even beyond the microtubule singlets. The ends of doublets, singlets, and central pair microtubules are each “capped” by ill-defined structures variously called caps or plugs^5^.

The distal tip of motile cilia in unicellular organisms has recently been resolved by CryoEM (Cryogenic electron microscopy). In *Tetrahymena* for example, the distal-most region of the axoneme is occupied only by the central pair, preceded by a region that also contains central pair and A-tubule singlets, with the normal 9+2 architecture finally arising about a micron from the tip^6,7^. Electron microscopy studies reveal that a similar configuration is conserved in *Chlamydomonas*^8–13^. The fine structure of the tips of motile cilia in vertebrate MCCs is less defined^14–16^, but studies of protein localization provide entry points for understanding.

For example, end-binding proteins that localize to MT plus ends such as Eb1 and Eb3 label the tips of both primary and motile cilia in vertebrates^17–20^. Other proteins label specifically the tips of motile cilia, for example the MT bundling protein Spef1 localizes to the central pair apparatus along the length of the axoneme^21^ but is dramatically enriched in the tips of *Xenopus*, mouse and human MCC cilia^20,22,23^. Other tip proteins described in unicellular organisms, such as Armc9 and Cep104^6,7,24^ have yet to be resolved clearly in vertebrate MCCs. How these proteins are recruited and arranged in distal cilia remains poorly defined.

Here, we provide a quantitative description of the molecular organization of the distal tips of vertebrate MCC cilia, revealing two distinct domains occupied by distinct protein populations. We find that the largely unstudied proteins Ccdc78 and Ccdc33 occupy a conserved, specialized domain in the extreme distal tip of MCC cilia in *Xenopus*, mouse, and human. Ccdc78 and Ccdc33 play non-redundant roles in maintaining both the normal localization of all other tip proteins and also for normal MCC cilia length control. These functions are specific to 9+2 cilia, and these proteins are neither present at the tip nor required for length control in other cilia types. Finally, *in vivo* and *in vitro* assays reveal that Ccdc78 and Ccdc33 act in concert to bundle microtubules. We conclude that Ccdc78/33 is a key module for organizing and stabilizing the architecture of the distal tip of motile cilia in vertebrate MCCs.

## Results

### Ccdc78 defines a novel domain at the extreme distal tip of MCC motile cilia.

To explore the cilia tip, we turned to the MCCs of *Xenopus* embryos (Fig. 1A), as they accurately reflect the biology of mammalian MCCs^25^, and their very large cilia make them highly amenable to imaging of protein localization^23,26^. We serendipitously found that a GFP-fusion to Ccdc78 specifically marked the tips of motile cilia in these cells (Fig. 1B). This fusion protein also marked deuterosomes, consistent with reports of its role in centriole amplification^27,28^ (Supp. Fig. 1A). We therefore confirmed localization at cilia tips by examining endogenous Ccdc78, which localized to the tip of human tracheal MCCs, as well as mouse airway and oviduct MCCs (Fig. 1C, D, E). In both *Xenopus* and human, Ccdc78 could be observed at the tips not only in full-length cilia, but also in shorter growing cilia at earlier stages (Supp. Fig. 1B, C, D)

We then compared the localization to Ccdc78 to that of microtubule bundling protein Spef1^29^, which we previously found enriched in the distal ends of MCC cilia in *Xenopus*, mice, and humans^20,22,23^. The two proteins displayed distinct patterns, with Spef1 labeling a broad domain and Ccdc78 highly restricted in the extreme distal tip (Fig. 1B, F). By quantifying pixel intensity with conventional confocal microscopy, we found that Spef1 was enriched in the distal-most two microns (Fig. 1F, magenta), but Ccdc78 was localized specifically within the final 0.5 microns of the same cilia (Fig. 1F, green).

This prompted us to examine localization using structured illumination super-resolution microscopy, which revealed that Spef1 and Ccdc78 report distinct domains, with Ccdc78 exclusively marking the distal-most 200nm of MCC cilia (Fig. 1G). Together, these data suggested that vertebrate MCC cilia tips are characterized by two distinct domains marked by Ccdc78 and Spef1.

### Quantification of the molecular organization of MCC cilia tips.

**Figure 2.**
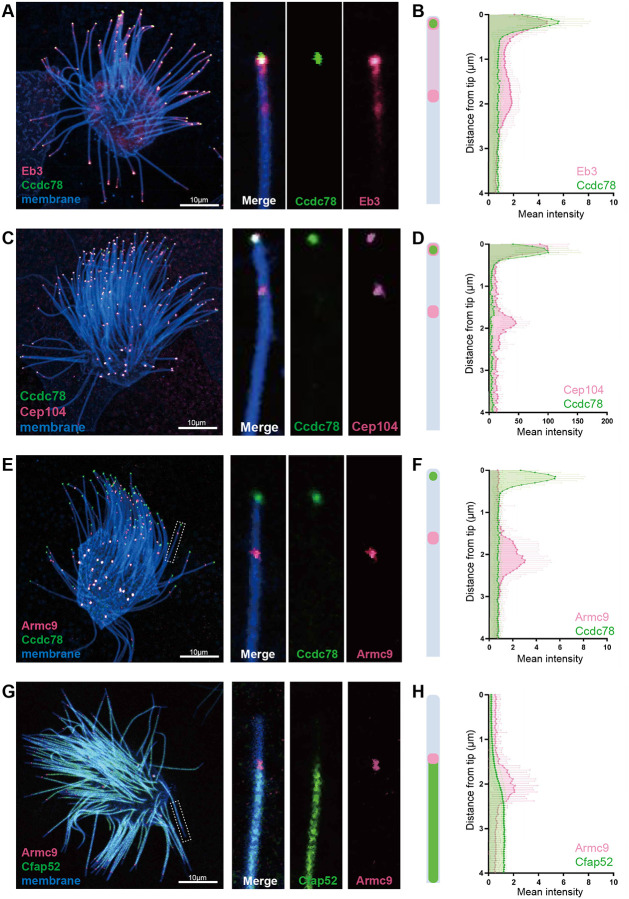
Ccdc78 localizes distally with known ciliary tip proteins in MCCs. (A) Localization of mScarlet3-Ccdc78 (green) with GFP-Eb3 (magenta), a single MCC is shown at left, with a single cilium from that cell shown at right. (B) Schematic image of result and quantification of pixel intensity for each protein in the distal-most 4 microns of cilia (see Methods). (C, D) Localization of mScarlet3-Ccdc78 (green) with GFP-Cep104 (magenta), with schematic and quantification. (E, F) Localization of GFP-Ccdc78 (green) with mScarlet3-Armc9 (magenta), with schematic and quantification. (G, H) Localization of mScarlet3-Armc9 (magenta) with GFP-Cfap52 (green), with schematic and quantification. (n=40 cilia for all graphs)

To further define the molecular organization of MCC cilia tips, we examined Eb3, as this microtubule end-binding protein localizes to the tips of both primary cilia and MCC cilia in *Xenopus* and humans^18,19^. Using conventional confocal microscopy, we observed diffuse enrichment of Eb3 that roughly overlapped with the Spef1 domain in the distal two microns of *Xenopus* MCC cilia (Fig. 2A). Unlike Spef1, however, the Eb3 intensity profile displayed two peaks, a minor peak around two microns from the tip and strong major peak in the final 0.5-microns, partially overlapping Ccdc78 (Fig. 2B).

To understand this pattern, we noted that in *Tetrahymena*, the distal tip of motile cilia is comprised only of central pair microtubules, with doublet microtubules terminating proximal to that region^30,31^. We reasoned that the two peaks of Eb3 localization may represent the end of the central pair at the extreme tip and the end of the doublets at two microns proximal. This interpretation is also consistent with the finding that Spef1 marks the central pair apparatus in vertebrate MCCs^21^ and specifically marks the distal ends of the central pair in *Tetrahymena*^6^.

As a further test of this idea, we next examined Cep104. This protein localizes to the tips of cilia as well as the basal body in mouse airway MCCs^32^, but in *Tetrahymena* localizes in two domains within the cilia tip, the plus ends of the doublets and of central pair^7^. Likely due to their large size, we discriminated two distinct domains of Cep104 localization in the tips of *Xenopus* MCC cilia, one at the extreme tip and another two microns proximally. Ccdc78 colocalized with Cep104 only in the extreme tip (Fig. 2C, D)

We then investigated Armc9, because this protein localizes to the distal ends of the B-tubules of the outer doublets in *Tetrahymena* motile cilia^30,33^, yet curiously localizes not to the tips but to the basal bodies in mammalian primary cilia^34 35^. In *Xenopus* MCCs, Armc9 was specifically enriched in a single, well-delineated domain, two microns proximal to the extreme distal tip (Fig. 2E, F, Magenta). Co-labeling with the B-tubule microtubule inner protein Cfap52 confirmed the positioning of Armc9 at the distal end of the B-tubule (Fig 2G, H)^36^ and suggests that Armc9 plays divergent roles in vertebrate primary and motile cilia. Thus, vertebrate MCC cilia tips display a highly ordered molecular architecture and Ccdc78 occupies a restricted region at the extreme distal end.

### Ccdc78 is required for localization of distal tip proteins in MCCs

**Figure 3.**
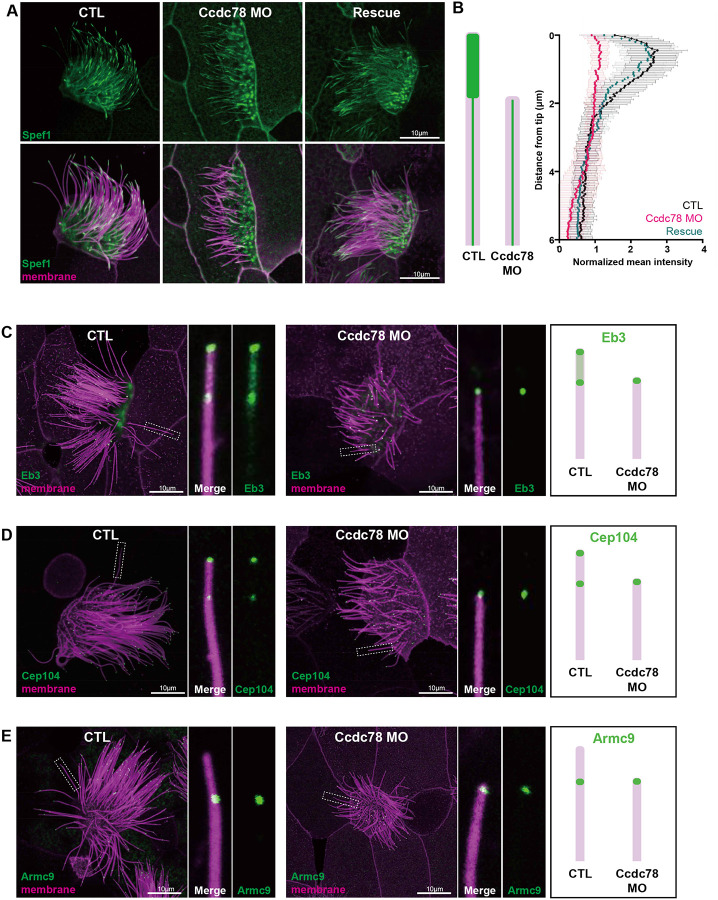
Ccdc78 is required for localization of distal tip proteins in MCCs (A) Image of GFP-Spef1 (green) and membrane-RFP (magenta) in *Xenopus* MCCs in control, Ccdc78 MO-injected or rescued embryos. Scale bars represent 10μm. (B) Schematic cartoon of Spef1 distribution in control and Ccdc78 MO-injected cilium. Quantification of fluorescent intensity of GFP-Spef1 along the axoneme was normalized by average intensity in control, Ccdc78 MO injected and rescued embryos. (n=58 cilia) (C-E) Localization of distal proteins in control and Ccdc78 MO injected embryos with magnified view and schematic cartoons on the right. Embryos were injected with Membrane-RFP (magenta) and GFP-Eb3 (C), GFP-Cep104 (D) or GFP-Armc9 (E) (green). Scale bars represent 10μm.

To explore the function of Ccdc78, we performed knockdown (KD) experiments by microinjecting anti-sense morpholino-oligonucleotides that efficiently disrupted the splicing of Ccdc78 (Supp. Fig. 2A). As a positive control, we confirmed that this KD significantly reduced the number of basal bodies in MCCs, consistent with its role in deuterosome function in the cytoplasm^28^ (Supp. Fig. 2B). More importantly, Ccdc78 KD also elicited severe disruption of Spef1 enrichment in the distal axoneme (Fig. 3A), a result we quantified using the scheme described above for Figure 2 (Fig. 3B). Importantly, the effect of Ccdc78 KD on both basal body numbers and Spef1 distal enrichment were rescued by re-expression of Ccdc78 (Fig. 3A, B; Supp. Fig. 2B), suggesting our KD is both effective and specific.

To better understand the impact of Ccdc78 KD, we next examined Eb3 localization, finding that the normally broad distribution with two peaks in the distal tip was eliminated. Instead, Eb3 marked only a single, restricted region at the very distal end of cilia after Ccdc78 KD (Fig. 3C; quantified in Supp. Fig 2C). Cep104 displayed a similar defect, with its two domains of localization collapsing to a single domain at the very tip after Ccdc78 KD (Fig. 3D; quantified in Supp. Fig 2D). Finally, the enrichment of the B-tubule end protein Armc9, normally present two microns proximal to the cilia tip was radically altered, marking the extreme distal ends of cilia after Ccdc78 KD (Fig. 3E; quantified in Supp. Fig 2E). We interpret these results together to mean that Ccdc78 KD elicits loss of the entire distal domain that is normally marked by Spef1 and Eb3 and normally extends two microns beyond the Armc9 domain. Thus, we conclude that after Ccdc78 KD, the central pair and doublet microtubules terminate together at the very end of cilia.

### Ccdc78 interacts physically and functionally with Ccdc33 in the MCC cilia tip

**Figure 4.**
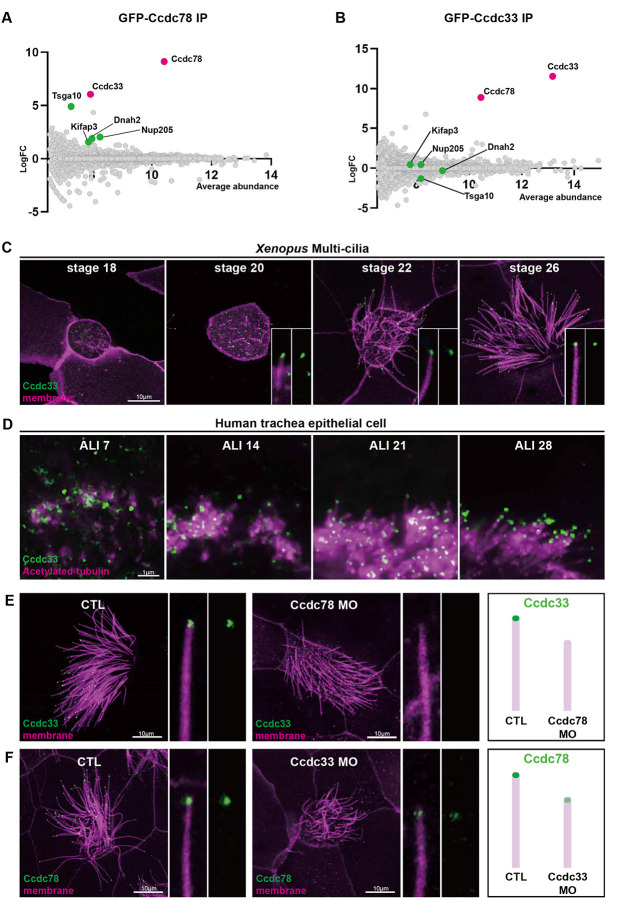
Ccdc78 interacts physically and functionally with Ccdc33 in the MCC cilia tip (A-B) MA plot of log2 fold change and average abundance in comparison between GFP and GFP-Ccdc78 (A) or GFP-Ccdc33 (B) immunoprecipitation enriched proteins. Highly enriched proteins are labeled with magenta (Ccdc78 and Ccdc33) and green (Tsga10, Nup205, Dnah2 and Kifap3). (C) Localization of GFP-Ccdc33 (green) with RFP-membrane (magenta) during ciliogenesis on *Xenopus* embryo epithelium. Magnified view of cilium on the bottom right. Scale bar represents 10μm. (D) Human tracheal epithelial cell multi-cilia stained with anti-acetylated tubulin (magenta) and anti-Ccdc33 (green) antibody during the time of ALI-culture day 7, 14, 21 and 28. Scale bar represents 1μm. (E) *Xenopus* MCC injected with membrane-RFP (magenta) and GFP-Ccdc33 (green) in control and Ccdc78 MO injected embryo with magnified view on the right side. Scale bar represents 10μm. Schematic cartoon is shown on the right. (F) *Xenopus* MCC injected with membrane-RFP (magenta) and GFP-Ccdc78 (green) in control and Ccdc33 MO-injected embryo with magnified view on the right side. Scale bar represents 10μm. Schematic cartoon is shown on the right.

Ccdc78 is a coiled-coil protein with no other domains to suggest its function. To gain new insights, we searched for interacting proteins using *in vivo* affinity purification from *Xenopus* MCCs and mass spectrometry (APMS). We expressed either GFP or GFP-tagged Ccdc78, isolated presumptive mucociliary epithelium, cultured until MCCs were fully developed^37,38^, and then performed APMS using anti-GFP beads (Supp. Fig. 3A). Compared to GFP alone, the most strongly enriched protein was the bait, Ccdc78, demonstrating the efficacy of the method (Fig. 4A).

Consistent with Ccdc78’s known role in the cytoplasm^27,28^, our APMS was also enriched for proteins acting at the base of cilia, including Nup205^39^ and Tsga10^40,41^ (Fig. 4A; Supp. Fig. 3B; Supp. Table 1). Other interactors were consistent with the axonemal localization for Ccdc78, such as the axonemal dynein Dnah2 and Kifap3, a subunit of the IFT kinesin (Fig. 4A; Supp. Table 1). Our APMS did not, however, identify any known vertebrate cilia tip proteins.

We therefore turned our attention to the most highly enriched protein, Ccdc33. We previously identified *ccdc33* as a direct target of the ciliary transcription factor Rfx2 and found it localized to axonemes^26,42^, but another group suggested the protein localizes to peroxisomes^43^. Nothing is known of Ccdc33 function. We first confirmed the interaction using reciprocal APMS with Ccdc33 as bait and found that Ccdc78 was the most strongly co-enriched protein (Fig. 4B). Cytoplasmic Ccdc78-interactors such as Nup205 and Tsga10 were not enriched by APMS for Ccdc33 (Fig. 4B; Supp. Table 2), suggesting that Ccdc33 and Ccdc78 act together specifically in the ciliary tip.

Consistent with that idea, GFP-Ccdc33 was not present at basal bodies, but rather localized specifically to ciliary tips at all stages of ciliogenesis in *Xenopus* MCCs (Fig. 4C; Supp. Fig. 4A). Endogenous Ccdc33 also labeled the tips of growing cilia in human tracheal epithelia cells differentiated in culture (Fig. 4D). Co-staining of human MCCs confirmed that endogenous Ccdc33 and Ccdc78 co-localize at cilia tips, while only Ccdc78 was also enriched in the apical cytoplasm (Supp. Fig. 4B).

**Figure 5.**
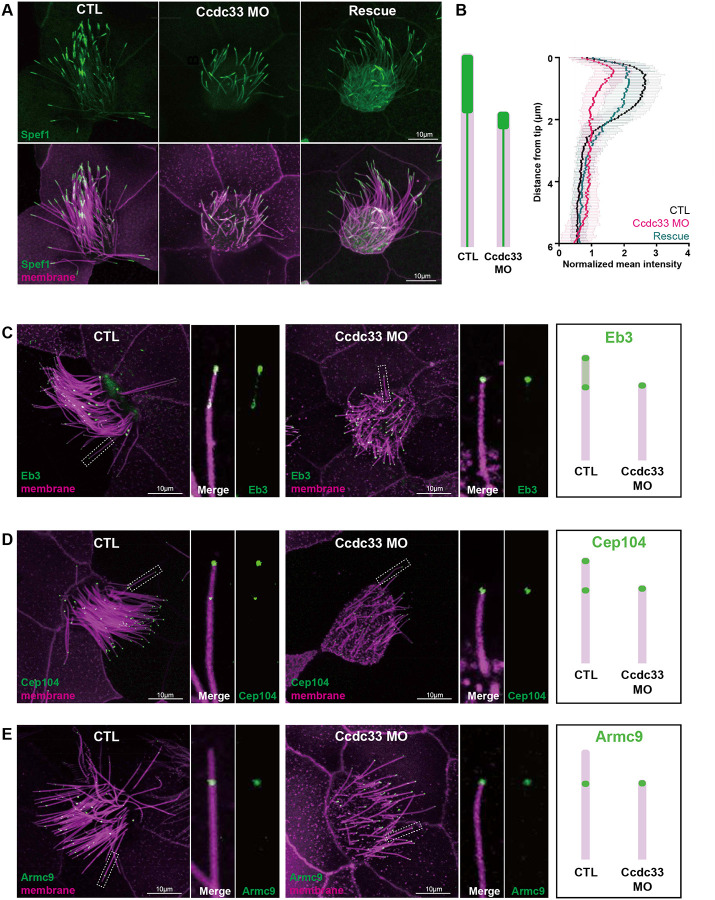
Ccdc33 is required for localization of distal tip proteins in MCCs (A) Image of GFP-Spef1 (green) and membrane-RFP (magenta) injected *Xenopus* multicilia in control, Ccdc33 MO-injected and rescued embryos. Scale bars represent 10μm. (B) Schematic cartoon of Spef1 distribution in control and Ccdc33 MO injected cilium. Quantification of fluorescent intensity of GFP-Spef1 along the axoneme normalized by average intensity in control, Ccdc33 MO-injected and rescued embryos. (n=65 cilia) (C-E) Localization of distal proteins in control and Ccdc33 MO-injected embryos with magnified view and schematic cartoons on the right. Embryos were injected with membrane-RFP (magenta) and GFP-Eb3 (C), GFP-Cep104 (D) or GFP-Armc9 (E) (green). Scale bars represent 10μm.

We then tested the functional inter-relationship of Ccdc78 and Ccdc33 at the tip of MCC cilia. Knockdown of Ccdc78 completely eliminated the localization of GFP-Ccdc33 in the distal axoneme (Fig. 4E; quantified in Supp. Fig. 5B). This KD did not reduce bulk protein levels of GFP-Ccdc33, suggesting a specific effect on localization (Supp. Fig. 5C). Reciprocally, we developed reagents for Ccdc33 KD, and these severely disrupted but did not eliminate the distal tip localization of Ccdc78 (Fig. 4F; quantified in Supp. Fig. 5D). Ccdc33 KD also elicited a reduction, but not a total loss, of the Spef1-enriched distal domain, and this effect was rescued by the expression of FLAG-tagged Ccdc33 mRNA (Fig. 5A, B), demonstrating the specificity of the KD.

Moreover, the effect of Ccdc33 KD on Eb3, Cep104, and Armc9 paralleled that of Ccdc78 KD, with loss of all elements that extend beyond the end of the microtubule doublets as marked by Armc9 (Fig. 5C–E; quantified in Supp. Fig. 6B–D). Unlike Ccdc78, however, Ccdc33 KD had no effect on basal body numbers (Supp. Fig. 6A), consistent with our proteomic and localization data suggesting Ccdc33 functions only at the tip and not in the cytoplasm. Thus, Ccdc78 and Ccdc33 act in concert and non-redundantly to control the molecular architecture of the distal tips of MCC cilia.

### Ccdc78 and Ccdc33 are essential for length control specifically in 9+2 motile cilia

**Figure 6.**
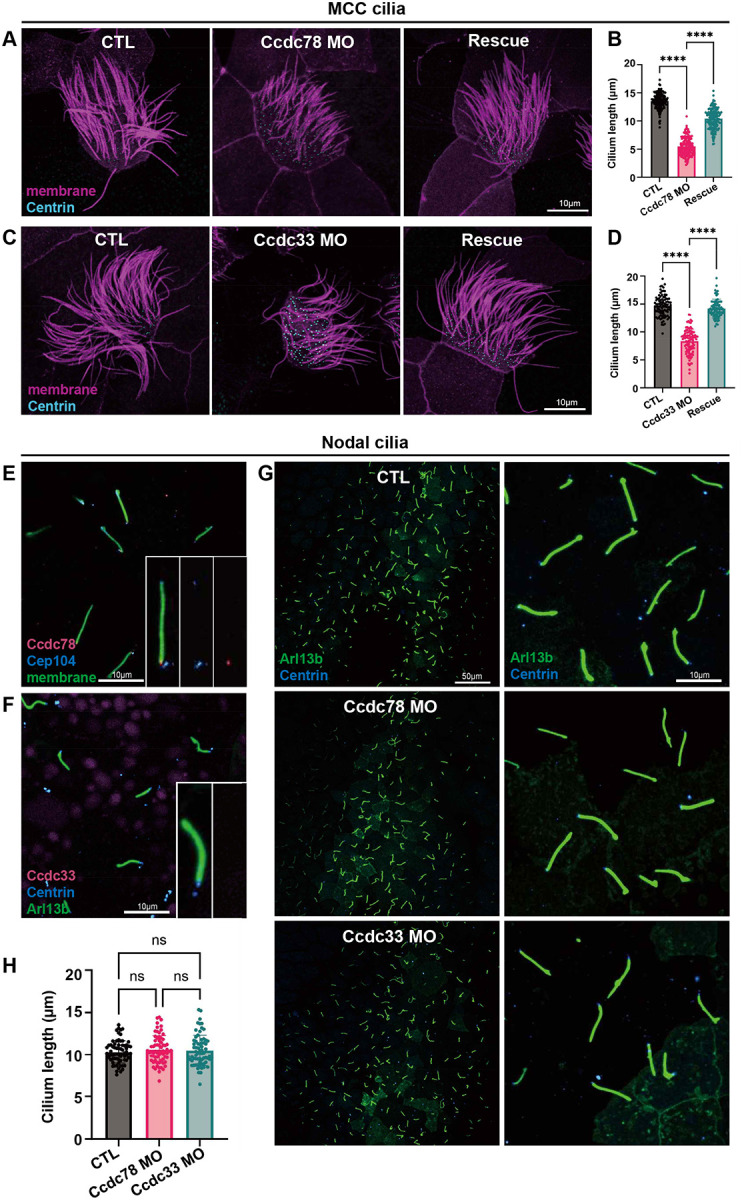
Ccdc78 and Ccdc33 are essential for length control specifically in 9+2 motile cilia (A) *Xenopus* MCC injected with membrane-RFP (magenta) and Centrin-BFP (cyan) in control, Ccdc78 MO and rescued embryos. Scale bar represents 10μm. (B) Quantification of the length of cilium. ****P<0.0001. (n=147 cilia) (Ordinary one-way ANOVA) (C) *Xenopus* MCC injected with membrane-RFP (magenta) and Centrin-BFP (cyan) in control, Ccdc33 MO and rescued embryos. Scale bar represents 10μm. (D) Quantification of the length of cilium is shown. ****P<0.0001. (n=100 cilia) (Ordinary one-way ANOVA) (E) Localization image of mScarlet3-Ccdc78 (magenta), GFP-Cep104 (blue) and membrane-BFP (green) in 9+0 motile cilia of *Xenopus* gastrocoel roof plate. Magnified view of cilium is inserted in bottom right. Scale bar represents 10μm. (F) Localization image of mScarlet3-Ccdc33 (magenta), GFP-Arl13b (green) and Centrin-BFP (blue) in 9+0 motile cilia. Magnified view of cilium is inserted in bottom right. Scale bar represents 10μm. (G) Image of *Xenopus* gastrocoel roof plate 9+0 motile cilia injected with GFP-Arl13b (green) and Centrin-BFP (blue) in control, Ccdc33 MO and Ccdc78 MO-injected embryos. Scale bar represents 50μm. Magnified view of images are shown in right with 10μm scale bar. (H) Quantification of the length of cilia. (n=68 cilia) (Ordinary one-way ANOVA)

Ccdc78 and Ccdc33 control localization of a coterie of proteins thought to stabilize the distal cilia tip, a region known to be important for ciliary length control. It was notable, then, that Ccdc78 KD severely reduced the length of MCC cilia. This phenotype was specific, since it was rescued by re-expression of Ccdc78 (Fig. 6A). Importantly, this shortening of cilia length could not be explained merely by the loss of the distal cilium, as the Spef1-positive domain spans only ~2 microns (~15% of cilium length) and the overall cilia length after Ccdc78 KD was reduced by >8 microns (~57% of cilium length) (Fig. 6B).

Ccdc33 KD also had a significant but more modest effect on cilia length; this effect too was specific since it was rescued by re-expression of Ccdc33 (Fig. 6C, D). Again, the loss of cilia length was too substantial (~6 microns) to be explained only by loss of the distal Spef1 domain. Thus, the extreme distal tip region occupied by Ccdc78 and Ccdc33 is essential for normal 9+2 cilia length regulation in MCCs.

To ask if Ccdc78/33 also control length in 9+0 motile cilia in the L/R organizer, we first examined their localization. While Ccdc78 was present at the base of motile cilia, it was not observed at the tips, which were labeled with Cep104 (Fig. 6E). Ccdc33 was not present at the base or the tip (Fig 6F). Moreover, KD of neither Ccdc78 nor Ccdc33 impacted the length of 9+0 motile mono-cilia in the *Xenopus* left/right organizer (Fig. 6G, H). Thus, Ccdc78 and Ccdc33 play non-redundant roles in assuring normal cilia length specifically in the 9+2 motile cilia of vertebrate MCCs (Supp. Fig 7).

### Ccdc78 and Ccdc33 display microtubule bundling activity *in vivo* and *in vitro*

Finally, we explored the mechanism of Ccdc78/33 action. The distal tips of motile cilia are subjected to high mechanical strain during beating^44,45^ and Spef1 is known to bundle microtubules^21,29^. We asked if Ccdc78 or Ccdc33 may have similar activity.

**Figure 7.**
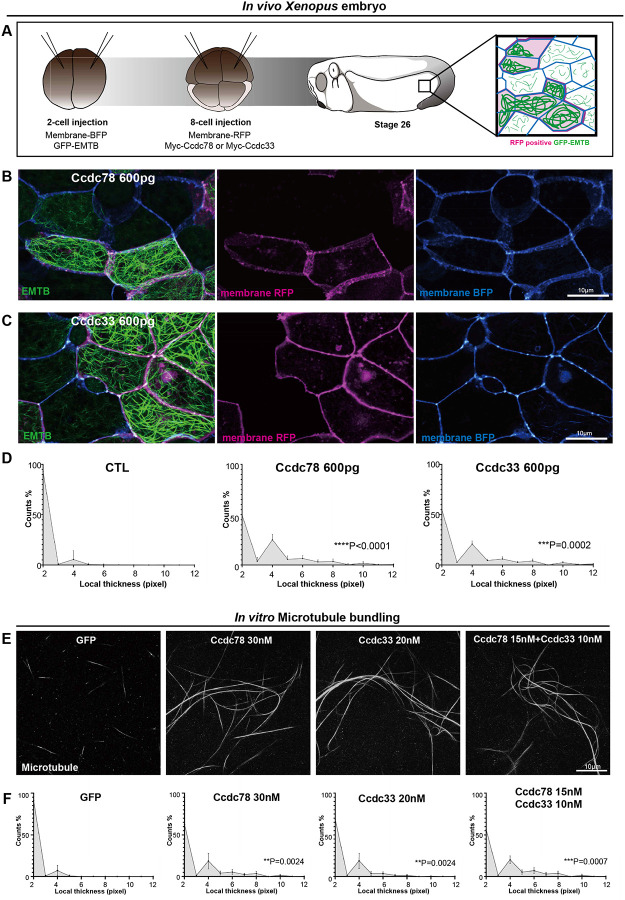
Ccdc78 and Ccdc33 show microtubule bundling activity *in vivo* and *in vitro* (A) Workflow for generating mosaic expression in *Xenopus* embryo. (B-C) Image of *Xenopus* embryo goblet cells on epithelium expressing GFP-EMTB (green), membrane-BFP (blue), and membrane-RFP(magenta) in embryos injected with Myc-Ccdc78 600pg (B) and Myc-Ccdc33 600pg (C). Cells expressing membrane-RFP correspond to those overexpressing Myc-ccdc78 or Myc-ccdc33, indicating mosaic expression. Scale bars represent 10μm. (D) Quantification of local thickness (n=26). The distribution of local thickness was compared between control and Ccdc78 600pg or Ccdc33 600pg using Kolmogorov-Smirnov test (KS-test), the KS-distance and P-value are shown in right. CTL vs Ccdc78 600pg ****P<0.005, CTL vs Ccdc33 600pg ***P=0.0002. (E) Image of 647-fluorophore labeled microtubules (gray) incubated with GFP, GFP-Ccdc78 30nM, GFP-Ccdc33 20nM and GFP-Ccdc78 15nM with GFP-Ccdc33 10nM. Scale bar represents 10μm. (F) Quantification of local thickness(n=15). The distribution of local thickness was compared between control and Ccdc78 600pg or Ccdc33 600pg using Kolmogorov-Smirnov test (KS-test), the KS-distance and P-value are shown in right. GFP vs GFP-Ccdc78 30nM **P=0.0024, CTL vs GFP-Ccdc33 20nM **P=0.0024, GFP vs GFP-Ccdc78 15nM & GFP-Ccdc33 10nM ***P=0.0007.

To this end, we uniformly labelled *Xenopus* embryo epidermis with the well-defined microtubule reporter (GFP-EMTB)^46–48^ and then made mosaics in which Ccdc78 was overexpressed in clones of cells marked with memRFP^49^ (Fig. 7A). We observed that memRFP labelled clones displayed robust bundling of microtubules that was not observed in cells outside the clone (Fig. 7B). Mosaic expression of Ccdc33 elicited the same effect (Fig. 7C). These increases in local thickness of microtubule bundles were highly significant (Fig. 7D). We also observed a similar phenotype with global rather than mosaic expression (Supp. Fig. 8A).

We then asked if Ccdc78 or 33 could bundle purified MTs *in vitro*. We co-incubated fluorescently labeled, polymerized, and Taxol-stabilized microtubules with *in vitro* translated GFP, GFP-Ccdc78, or GFP-Ccdc33. MTs incubated with GFP alone formed only sparse, small bundles. By contrast, the addition of either Ccdc78 or Ccdc33 resulted in the formation of prominent bundles throughout the sample (Fig. 7E, F).

Finally, we asked if the two proteins might function in concert. When provided alone, 30nM Ccdc78 or 20nM Ccdc33 were sufficient to achieve robust bundling. However, in combination, 15nM and Ccdc78 together with 10nM Ccdc33 were also able to induce bundling to a similar degree (Fig. 7E, F). These results demonstrate that Ccdc78 and Ccdc33 possess microtubule bundling activity *in vivo* and *in vitro* and support the idea that the two proteins work in concert to stabilize the distal tip of MCC motile cilia.

## Discussion:

We show here that Ccdc78 and Ccdc33 occupy a distinct region of the extreme distal tip of vertebrate MCC motile cilia. They function non-redundantly there and are required for normal localization of several additional ciliary tip proteins. Moreover, Ccdc78 and Ccdc33 – and by extension of the extreme distal tip region – are necessary for normal ciliary length. Finally, Ccdc78 and Ccdc33 display robust microtubule bundling activity both *in vivo* and *in vitro*. The data provide a new depth of understanding of the molecular architecture of MCC cilia tips and are significant on several levels.

First, it has been frequently noted that the structure of distal tips of motile cilia are highly variable across species and cell type^5^, yet our data suggest that the molecular architecture underlying these structures is largely shared. Indeed, our data reveal that Spef1, Cep104, and Armc9 in the distal tips of *Xenopus* MCC cilia display similar patterns to that observed in *Tetrahymena*^6,7,24^. Likewise, vertebrate Ccdc33 combines coiled-coil regions with lipid-binding C2 domains, so may be a direct orthologue of the similarly structured C2D1 protein (Uniprot: I7LU65) reported at the distal ends of *Tetrahymena* cilia^6,7^(Supp. Fig. 9A–C). Curiously, a protein with homology to Ccdc78 has also been found at the distal tip of *Tetrahymena* cilia (Uniprot: I7LVY1)^7^, but that protein is characterized by a kinesin motor domain absent from vertebrate Ccdc78 (Supp. Fig. 9D–F). Regardless, our data reveal the conservation of molecular architecture in the tips of motile cilia that is unexpected given the divergent fine structures.

Second, our data suggest two activities for Ccdc78/33 which are not mutually exclusive. Because these proteins are sufficient to bundle microtubules (Fig. 7), Ccdc78/33 likely play direct roles in stabilizing the microtubules of the distal axoneme. The loss of Cep104, Spef1 and Eb3 from ciliary tips in the absence of Ccdc78/33 (Fig. 3, 5) may be a secondary effect of destabilizing the tip, an idea bolstered by the reduction of ciliary length (Fig. 6). Alternatively, Ccdc78/33 may also serve a scaffolding function, somehow organizing the recruitment of those other proteins independently of their role in MT bundling. Because neither our APMS (Supp. Tables 1, 2) nor extensive testing with AlphaFold^50,51^ (not shown) identified interaction between Ccdc78/33 and any known tip proteins, the former possibility may be more likely.

The defects in ciliary length observed after loss of Ccdc78 or 33 are also interesting. Since they cannot be explained simply by loss of the distal tip region (i.e. that marked by Spef1), our data suggest two possibilities. First, these axonemes are destabilized in the absence of Ccdc78/33, and in turn are shortened by disassembly as a consequence of the forces associated with ciliary beating. Indeed, the whip-like movement of cilia increases mixing and velocity of the surrounding fluid most strongly near ciliary tips^44,45^, suggesting the distal axoneme is subjected to higher mechanical stress. In this light, it is notable that like Ccdc78/33, Spef1 also displays robust MT bundling and stabilizing activity *in vivo* and *in vitro*^52–54^. Alternatively, since elongation of the axoneme is controlled at the tip, Ccdc78/33 may control the elongation machinery directly. It is interesting in this light that ciliopathy patients with mutation in Armc9 display destabilized non-motile primary cilia^35^.

Finally, the activity of Ccdc78/33 are consistent with mechanical stability being a key role of the distal tip specializations in MCCs. Indeed, Ccdc78 and 33 do not localize to the tips of rotationally beating 9+0 motile cilia at the left/right or primary cilia (Fig. 6E, F). This is in direct contrast to other elements of cilia tips such as Eb1 and Eb3 that are present at the tips of all cilia^55,56^. Thus, stabilization is a cell type-specific, essential function in MCC cilia. How this function relates to other cell-type specific functions at the distal tip (e.g. ectosome shedding^57,58^) and how it relates to universal cilia tip functions (e.g. IFT turnaround^59–61^) is an important open question. Nonetheless, we did identify an IFT kinesin subunit as a Ccdc78 interactor (Kif3ap).

Finally, our data suggest that Ccdc33 and Ccdc78 are candidate genes for distinct variants of human motile ciliopathy. Some patients with primary ciliary dyskinesia display airway defects without associated situs anomalies, suggesting a specific defect in MCC cilia. Because Ccdc33 loss disrupts MCC cilia tips specifically and is not present in the cytoplasm, it is worth consideration as a candidate gene for patients with this disease. By contrast, more severe airway disease results from defects in transcription factors that control centriole duplication^62^, and because Ccdc78 is essential for both cilia tip organization (Fig. 3, 6) and basal body duplication in MCCs (Supp. Fig. 2B)^28^, this gene may be a candidate for more severe airway ciliopathies. Our APMS implicates other basal body proteins like Nup205 and Tsga10 in Ccdc78 function in the cytoplasm, which may also inform those diseases. Thus, Ccdc78 and Ccdc33 represent an important module acting at the extreme tip of motile cilia to control cilia length. As uch, these genes represent interesting candidate loci for motile ciliopathy.

## Materials and Methods

### Animal husbandry and embryo manipulation

To induce the ovulation, female adult *Xenopus* laevis was injected with 400unit of hCG (Human chorionic gonadotropin) and incubated in 16°C incubator for overnight. In vitro fertilization was performed by mixing the *Xenopus egg* with homogenized testis in 1/3X MMR (Marc’s Modified Ringer’s). Fertilized embryos were then de-jellied with 3% L-cysteine in 1/3X MMR (pH.7.8), washed and manipulated in 1/3X MMR.

### Plasmids, mRNA and Morpholino

Gene sequence for *Xenopus laevis* was obtained from Xenbase (https://www.xenbase.org/). Total RNA was purified from *Xenopus laevis* embryo and then reverse transcribed into cDNA library using M-MLV Reverse Transcriptase (Invitrogen). Coding sequence (CDS) of genes were amplified from the *Xenopus* cDNA library by PCR (Polymerase chain reaction) using Q5^®^ High-Fidelity DNA Polymerase (NEB). Primer pairs were designed with restriction enzymes sites for cloning. Amplified genes and pCS10R vector containing fluorescence tag were digested with restriction enzymes, ligated together and inserted into competent cells by transformation. Cloned constructs were linearized to synthesize mRNA using mMESSAGE mMACHINE^™^ SP6 Transcription Kit (Invitrogen). The list of cloned genes are as follows: Armc9, Cep104, Ccdc78, Ccdc33, Spef1, Spag6, Cfap52, Ak7, Tsga10, EMTB.

Anti-sense morpholinos were designed to block RNA splicing based on the sequence from Xenbase database. The morpholinos were manufactured by Gene Tools. Ccdc78 and Ccdc33 morpholino sequences are as follows:

Ccdc78.L MO: 5’-CCCATTCCTTTCACTTACATTTTC-3’
Ccdc33.L MO: 5’-GGTCAGGTAGTCACAGTATAAGAA-3’

### Embryo microinjection and sample preparation

Embryo microinjection was done in 2% ficoll in 1/3X MMR. For the multi-cilia visualization, 2-cells of ventral-animal side in 4-cell stage embryos were injected with fluorescence tagged mRNA and morpholinos. For the nodal cilia visualization, two dorsal-ventral-cells in 4-cell stage embryos were injected with GFP-tagged Arl13b, BFP-tagged cent4 and mScarlet3-tagged Ccdc78 or Ccdc33.

For multi-ciliated cells live-imaging, *Xenopus* embryos were incubated until stage 26 after microinjection. Whole embryos were mounted between the cover glass with a small amount of 1/3X MMR and imaged immediately. For nodal cilia live-imaging, *Xenopus* embryos were incubated until stage 18 after the microinjection. Embryos were dissected laterally to dorsal-posterior to obtain the gastrocoel roof plate (GRP) region. Dissected explants were mounted in the same way as whole embryos and imaged immediately.

### Image acquisition and analysis

Confocal images were acquired with Zeiss LSM700 laser scanning confocal microscope using a plan-apochromat 63X/1.4 NA oil objective lens (Zeiss). Structured illumination microscopy (SIM) images were acquired with Nikon DeepSIM microscopy using a 100X oil objective lens (Nikon).

Quantitative measurement of images was done using Fiji. The fluorescent intensity values of each measured pixel for each protein was normalized with the average intensity of the entire distal-most four microns of individual cilia. Graph generation and statistical analysis including error bars and mean ± SD and P values were performed using Prism 10 software.

### Protein purification and Immunoprecipitation

GFP-tagged Ccdc78 and Ccdc33 plasmid respectively were injected into 2-cell of ventral-animal side of the *Xenopus* embryos in 4-cell stage. Injected embryos were incubated until they reached stage 9, and their animal caps were dissected in Steinberg’s solution with gentamicin. When animal caps reached stage 26, animal caps were collected for protein extraction.

Protein purification and immunoprecipitation were performed with GFP-Trap agarose kit (Chromotek). Collected embryos were lysed with lysis buffer containing 1X protease inhibitor (Thermo Scientific^™^ Halt^™^ Protease Inhibitor Cocktail (100X), Thermofisher) and 1mM PMSF (Phenylmethylsulfonyl fluoride). Lysed samples were centrifuged in 4°C for 15 minutes with 14000rpm to separate the lipid layer and collect clear lysates. GFP-agarose beads were equilibrated in dilution buffer according to the manufacturer’s protocol. GFP-agarose beads in dilution buffer were added to lysate and incubated at 4°C for 1 hour. After protein binding, beads were centrifuged at 4°C for 5 minutes with 2500xg. Pelleted beads were washed with wash buffer 2 times and added in 1.5X Laemmli sample buffer with 5% (v/v) 2-Mercaptoethanol. After boiling at 95°C, beads were pelleted with 4°C centrifuge for 5 minutes and the supernatant was collected and stored at −80°C for further analysis.

### Affinity purification Mass spectrometry

Immunoprecipitated proteins were resuspended in SDS-PAGE sample buffer and heated 5 min at 95°C before loading onto a 7.5% acrylamide mini-Protean TGX gel (BioRad). After 7 min of electrophoresis at 100 V the gel was stained with Imperial Protein stain (Thermo) according to manufacturer’s instructions. The protein band was excised, diced to 1 mm cubes and processed for in-gel trypsin digestion as in Goodman et al., 2018 (PMID: 30259661, PMCID: PMC6492177, DOI: 10.1002/pmic.201800236). Digested peptides were desalted with 6μg-capacity ZipTips (Thermo Scientific), dried, and resuspended in 20μl of 5% acetonitrile, 0.1% acetic acid for mass-spectrometry. Peptides were separated using reverse phase chromatography on a Dionex Ultimate 3000 RSLCnano UHPLC system (Thermo Scientific) with a C18trap to EASY-Spray PepMap RSLC C18 column (Thermo Scientific, ES902) configuration eluted with a 3% to 45% gradient over 60 min. Spectra were collected on a Thermo Orbitrap Fusion Lumos Tribrid mass spectrometer using a data-dependent top speed HCD acquisition method with full precursor ion scans (MS1) collected at 120,000 m/z resolution. Monoisotopic precursor selection and charge-state screening were enabled using Advanced Peak Determination (APD), with ions of charge   + two selected for high energy-induced dissociation (HCD) with stepped collision energy of 30% +/− 3% (Lumos) Dynamic exclusion was active for ions selected once with an exclusion period of 20 s (Lumos). All MS2 scans were centroid and collected in rapid mode. Raw MS/MS spectra were processed using Proteome Discoverer (v2.5) and the Percolator node to assign unique peptide spectral matches (PSMs) and protein assignments (FDR .01) to a X. laevis proteome derived from the 2023 UniProt Xenopus laevis reference proteome of 35,860 protein sequences with homeologs and highly related entries collapsed into EggNOG vertebrate-level orthology groups (Huerta-Cepas et al., 2016). This database and the mass spectrometry data are available on MassIVE https://massive.ucsd.edu/ProteoSAFe/static/massive.jsp (MSV000096822).

In order to identify proteins significantly associated with each bait, we used the degust statistical framework (https://degust.erc.monash.edu/) to calculate both a log2 fold-change and an FDR for each protein enrichment based on the observed PSMs in the bait versus control pulldown. Settings used were “RUV (edgeR-quasi-likelihood), Normalization TMM, and Flavour RUVr” and at least 2 counts in at least 2 samples.

### RT-PCR

To confirm the efficacy of Ccdc78 and Ccdc33 morpholinos, morpholinos were injected into all cells at 4-cell stage of embryo. Total RNA was isolated with Trizol reagent at stage 26, and cDNA was synthesized with M-MLV reverse transcription kit (Invitrogen). Ccdc78, Ccdc33 and Odc1 were amplified by Taq-polymerase (Invitrogen) with the primers as follows:

Ccdc78 Forward: 5’-AGAAGATCGTGAGACCCCCC-3’
Ccdc78 Backward: 5’-TCAAGCTCTGCTGTGGCTTC-3’
Ccdc33 Forward: 5’-GCTTCCAGAACAACACCTACCTTT-3’
Ccdc33 Backward: 5’-GTGAGACAGGCGGAGAATCATCTA-3’

### Immunoblotting

*Xenopus laevis* embryos were injected with GFP-Ccdc78 or GFP-Ccdc33 in all cells at 4-cell stage with Ccdc33 or Ccdc78 morpholino, respectively. At stage 26, embryos were lysed in lysis buffer containing 1X protease inhibitor (Thermo Scientific^™^ Halt^™^ Protease Inhibitor Cocktail (100X), Thermofisher) and 1mM PMSF (Phenylmethylsulfonyl fluoride). Fat was removed by centrifuging the samples with 12000xg in 4°C for 15 minutes. The clear lysate was added with 4X Laemmli sample buffer with 5% (v/v) 2-Mercaptoethanol and heated to 95°C for 10 minutes. The protein samples were further processed for the SDS-PAGE and immunoblotting as described below.

Protein samples were loaded on SDS-PAGE and transferred to nitrocellulose membranes. The membrane was incubated in blocking solution (0.05% Tween-20 in TBS with non-fat powdered milk) at room temperature for 30 minutes. For immunoblotting, membranes were sealed with primary antibodies in 1% BSA solution for 1 hour at room temperature. Secondary labeling was performed using horseradish peroxidase (HRP)-conjugated secondary antibodies for 1 h at room temperature. Chemiluminescence was performed with enhanced chemiluminescence substrate and imaged with Image Quant (LAS 4000, GE Healthcare).

The primary antibodies used were as follows:

mouse anti-beta-actin (1:10000, 66009-1, Proteintech); mouse anti-GFP (1:1000, sc-9996, Santacruz Biotechnology).

The secondary antibodies used were as follows:

Anti-mouse IgG HRP-conjugated (1:2000, 31430, ThermoFisher Scientific)

### Human ALI culture and immunostaining

Human trachea epithelial cells (HTECs) culture were prepared from airway epithelial cells isolated from tracheobronchial tissues as previously described^63^. Use of human tissues was reviewed by the Washington University Institutional Review Board. Anonymized human tissues were obtained from surgical excesses of deceased individuals and experimented by Code of Federal Regulations, 45 CFR Part 46, as not meeting criteria for human subject research. Briefly, epithelial cells were isolated from the tissues following incubation in pronase and differential adhesion on tissue culture plates. Basal cells were expanded in custom media, then released by trypsin treatment and cultured on Trans-well membranes (Trans-well, Corning, 3460). Cells were differentiated using air-liquid interface (ALI) conditions and media previously described^63^.

Cultured airway cells were immunostained in situ on the Trans-well membranes. Cells were fixed with 4% paraformaldehyde (PFA) for 10 min at room temperature. Non-specific antibody binding was blocked in buffer containing 3% bovine serum albumin and 0.1% TritonX100 in PBS for 1 hour at room temperature. Cells were then washed with 0.1% Tween20 in phosphate buffer saline (PBS) and incubated in primary antibodies diluted in blocking buffer. Primary antibodies were anti-CCDC78 (1:100, Proteintech, 26876–1-AP), anti-CCDC33 (1:100, Santacruz, sc-390852) and acetylated alpha tubulin (mouse, Clone 6–11B- 1, 1:20.000; Sigma-Aldrich, Cat# T7451; RRID: AB_609894). After incubation at 4 °C overnight, the cells were then washed and incubated with species-specific, fluorescent-labeled secondary antibodies diluted in PBS for 45–60 min at room temperature. Membranes were mounted on glass slides in medium containing 4’, 6-diamidino-2-phenylindole (DAPI) to stain DNA (Fluoroshield, Sigma Aldrich, # F6057). Cells were imaged by wide-field fluorescent microscopy using an upright Leica 6000 microscope equipped with a cooled digital camera interfaced with imaging software (LAS X, Leica).

### Mouse tissue immunostaining

The trachea and oviduct were collected from P21 mouse. Formalin-fixed and paraffin-embedded (FFPE) tissues were sectioned at 4 μm. The FFPE sections were dewaxed, rehydrated, and boiled in antigen retrieval solution Histo-VT-One (Nacarai). After blocking with Blocking-One-Histo (Nacarai), the sections were incubated with anti-CCDC78 antibody (1:100, Proteintech, 26876–1-AP) and acetylated-tubulin (1:300, Proteintech, 66200–1-Ig) overnight at 4 °C, then with Alexa Fluor-conjugated secondary antibodies (1:500, Invitrogen, A-21206, A-21203). The sections were mounted in Fluorescence-Mounting-Medium (Dako).

Immunofluorescence imaging was performed on a LSM800 confocal laser scanning microscope (Carl Zeiss).

### *In vitro* microtubule bundling

*In vitro* translation of GFP, GFP-Ccdc78 and GFP-Ccdc33 proteins were performed using TnT^®^ Coupled Wheat Germ Extract System (Promega). Briefly, 2μg of CS10R-GFP, CS10R-GFP-Ccdc78 and CS10R-GFP-Ccdc33 plasmids were incubated with 30μl of TnT^®^ SP6 High-Yield Wheat Germ Master Mix in total 50μl reaction, 25°C 2 hours. The concentration of proteins was determined with western blot analysis by comparing translated proteins to serial dilutions of GFP peptide.

To polymerize microtubules *in vitro*, 2μl of 647-labeled tubulins (2mg/ml)(Pursolutions, 064705) were diluted in 6μl of 1mM GTP containing tubulin buffer (80mM PIPES pH 6.9, 1mM EDTA, 1mM MgCl2, 10% glycerol). Tubulins were polymerized for 1 hour in 37°C water bath and stabilized by adding 35μM of Taxol solution to the polymerized microtubules. After an additional 20 minutes incubation in 37°C water bath, microtubules were stored at room temperature covered with foil to avoid light.

For microtubule bundling assay, stabilized microtubules were diluted into tubulin buffer in 1:20 dilution. 6μl of diluted microtubules were mixed with 4μl GFP, 4μl GFP-Ccdc78, 4μl GFP-Ccdc33 and 2μl GFP-Ccdc78 + 2μl GFP-Ccdc33. After incubation at room temperature for 20 minutes, 10μ l of each mixture was gently squashed between slide glass and coverslip and sealed with VALAP (1:1:1 Vaseline: lanolin: paraffin wax).

### Alphafold3 structure prediction

The protein structure predictions were predicted by Alphafold3 using Alphafold server (https://alphafoldserver.com/).

## Supplementary Material

Supplement 1 **Supplemental Table 1:** APMS data for Ccdc78 Pulldowns. Raw data available on MassIVE https://massive.ucsd.edu/ProteoSAFe/static/massive.jsp (MSV000096822).

Supplement 2 **Supplemental Table 2:** APMS data for Ccdc33 Pulldowns. Raw data available on MassIVE https://massive.ucsd.edu/ProteoSAFe/static/massive.jsp (MSV000096822).

1 **Supplementary figure 1. Localization of Ccdc78 in MCC cytosol during ciliogenesis** (A) Image of *Xenopus* MCC cytosol expressed with GFP-Ccdc78 (green) and Centrin-BFP (gray) in stage 18, 20 and 26 embryos. Localization of GFP-Ccdc78 on the basal body of MCC is shown on the right. Scale bars represent 10μm.
(B) Schematic cartoon of localization of Ccdc78 during multi-ciliogenesis.
(C) Apical surface of *Xenopus* embryo epithelium of stage 18, 20, 22, and 26, showing the localization of GFP-Ccdc78 (green) with Membrane-RFP (blue). Insets show magnified views of cilia. Scale bars represent 10μm.
(D) HTEC multi-cilia stained with anti-acetylated tubulin (magenta) and anti-Ccdc78 (green) antibody during the time of ALI-culture day 7, 14, 21 and 28. Scale bar represents 1μm.
**Supplementary figure 2. RT-PCR of Ccdc78 splice-blocking morpholino-injected embryos and quantification of distal protein distributions** (A) Gel image of RT-PCR showing *ccdc78* and *odc1* mRNA levels in control embryos and embryos injected with 10 ng or 20ng of Ccdc78 MO.
(B) Image of basal bodies expressed with Centrin-BFP (cyan) in control, Ccdc78 MO and rescue embryos. Scale bar represents 10 μm. Quantification of the number of basal bodies per cell is shown on the right. ****P<0.0001, **P<0.01. (n=31 cells) (Ordinary one-way ANOVA).
(C-E) Quantification of the normalized mean intensity distribution of distal proteins Eb3 (C), Cep104 (D) and Armc9 (E) shown in Fig. 3C, D, E. (n=82 cilia)
**Supplementary figure 3. AP-MS performed using *Xenopus* animal caps and localization of Tsga10 in *Xenopus* MCC** (A) Workflow for AP-MS performed using *Xenopus* animal caps.
(B) Localization of GFP-Tsga10 (green) with membrane-BFP (blue) and Ccdc78 (magenta) in *Xenopus* MCC. Scale bar represents 10μm.
**Supplementary figure 4. Localization of Ccdc33 in *Xenopus* MCC cytosol and HTEC** (A) Images of *Xenopus* MCC cytosol expressed with GFP-Ccdc33 (green) and Centrin-BFP (gray) in stage 18, 20 and 26 embryos. The localization of GFP-Ccdc33 on the basal body of MCC is shown on the right. Scale bars represent 10μm.
(B) HTEC stained with anti-Ccdc78 (magenta) and anti-Ccdc33 (green) antibodies in multi-cilia. Scale bar represents 1μm.
**Supplementary figure 5. Validation of Ccdc33 splice-blocking morpholino efficiency by RT-PCR and distribution of Ccdc78/Ccdc33 intensity along the cilia after Ccdc33/Ccdc78 MO injection** (A) Gel image of RT-PCR of *ccdc33* and *odc1* mRNA levels in control, Ccdc33 MO 5ng and 7ng injected embryos.
(B-E) Schematic cartoon with distribution of GFP-Ccdc33 (B) or GFP-Ccdc78 (D) intensity along the axoneme after Ccdc78 MO (B) or Ccdc33 MO (D) injection. (n=72 cilia). The total amount of GFP-Ccdc33 (C) or GFP-Ccdc78 (E) proteins was confirmed by western blot using anti-GFP and anti-beta-actin as a loading control.
**Supplementary figure 6. Knockdown effect of Ccdc33 on basal bodies and distal tip proteins in *Xenopus* MCC** (A) Image of basal bodies expressed with Centrin-BFP (cyan) in control, Ccdc33 MO-injected embryos. Scale bar represents 10μm. Quantification of the number of basal bodies per cell is shown on the right. P=0.2066. (n=30 cells) (Unpaired t-test).
(B-D) Quantification of normalized mean intensity distribution of distal proteins Eb3 (B), Cep104 (C) and Armc9 (D) shown in Fig. 5C–E. (n=65 cilia)
**Supplementary figure 7. Ccdc78 and Ccdc33 specifically affect 9+2 motile cilia in** MCC (A-B) Schematic images of *Xenopus* embryo at tailbud stage (A) and neurula stage (B). The MCC 9+2 cilia are composed of microtubule doublets and a central pair where the extreme distal tip of central pair is labeled with Ccdc78/33. Loss of Ccdc78/33 results in disruption of distal ciliary region in MCC. On the other hand, nodal 9+0 cilia are composed of microtubule doublets only. Ccdc78/33 is not present in nodal cilia tip, thereby the 9+0 motile cilia are not affected by Ccdc78/33 deficiency.
**Supplementary figure 8. Mosaics on *Xenopus* embryo epithelium and Western blots of Myc-Ccdc78 and Myc-Ccdc33** (A) Image of *Xenopus* embryo goblet cells on epithelium expressed with GFP-EMTB (green), membrane-RFP (magenta) and membrane-BFP (blue). Scale bar represents 10μm.
(B-C) Western blots to validate ectopic expression of Myc-Ccdc78, Myc-Ccdc33, and GFP-EMTB with Beta-actin used as a loading control.
**Supplementary figure 9. Structural differences of Ccdc78 and Ccdc33 in *Tetrahymena* and *Xenopus laevis*** (A) Structural domain of *Xenopus* Ccdc33 and *Tetrahymena* I7LU65.
(B-C) Alphafold prediction of protein structures for *Xenopus* Ccdc33(B) and *Tetrahymena* I7LU65(C) with a 90-degree rotation. Coiled-coil regions are colored light blue, intrinsically disordered regions (IDRs) are colored yellow and C2 domains are colored dark blue.
(D) Structural domain of *Xenopus* Ccdc78 and *Tetrahymena* I7LVY1.
(E-F) Alphafold prediction of protein structures for *Xenopus* Ccdc78(E) and *Tetrahymena* I7LVY1(F). Coiled-coil regions are colored gray, and the kinesin motor domain is colored green.
