# Supplementary material for: A protein complex in the extreme distal tip of vertebrate motile cilia controls their organization, length, and function
Source: bioRxiv. 2025 Jun 25:2025.02.19.639145. Originally published 2025 Feb 19. Preprint. [Version 2] doi: 10.1101/2025.02.19.639145 (PMC11870508; doi:10.1101/2025.02.19.639145)

bioRxiv preprint doi: https://doi.org/10.1101/2025.02.19.639145; this version posted June 25, 2025. The copyright holder for this preprint (which was not certified by peer review) is the author/funder, who has granted bioRxiv a license to display the preprint in perpetuity. It is made available under aCC-BY 4.0 International license.

# Supplementary Figure 1. Localization of Ccdc78 in *Xenopus* MCC and human airway cells along the developmental stages

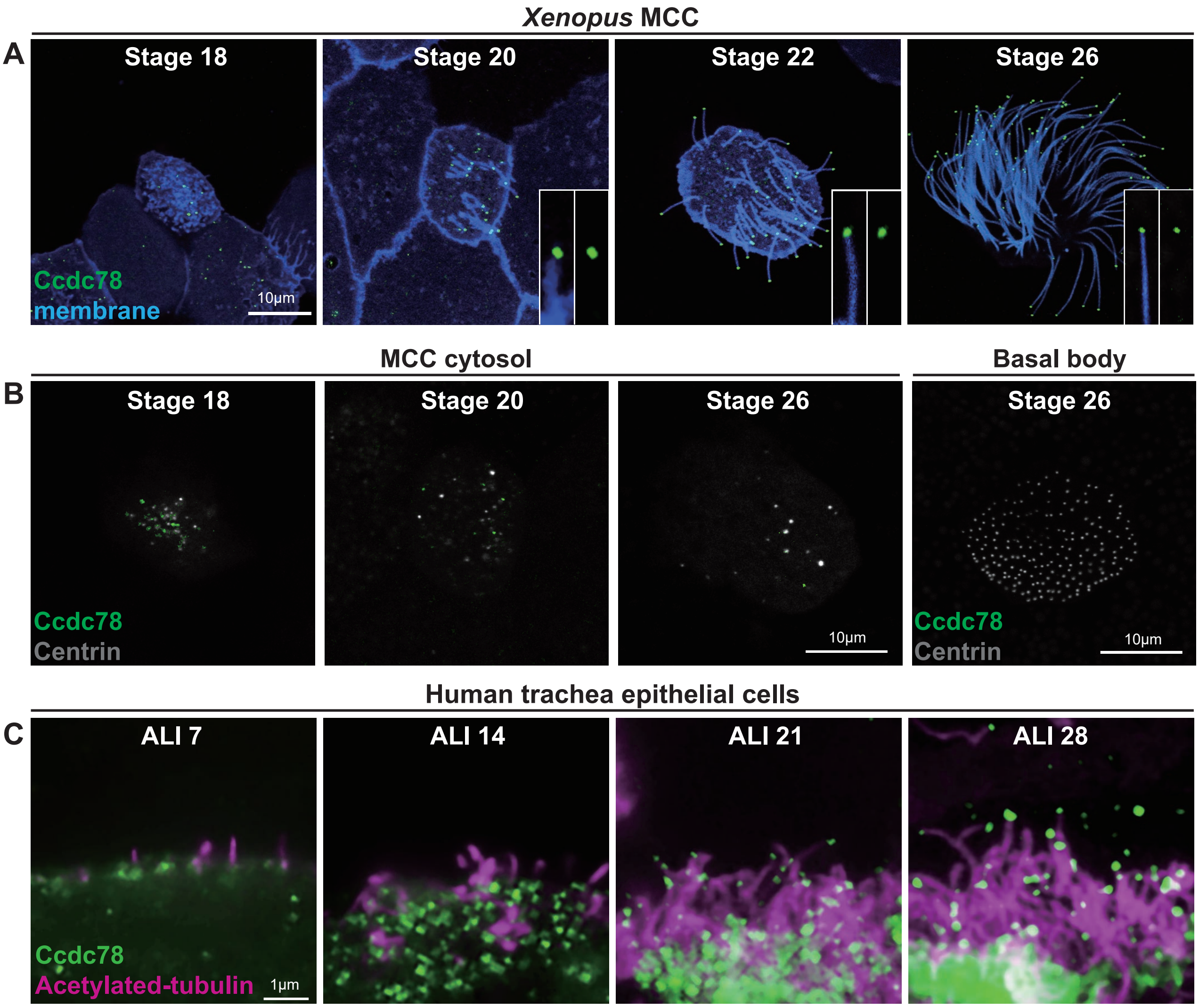

bioRxiv preprint doi: https://doi.org/10.1101/2025.02.19.639145; this version posted June 25, 2025. The copyright holder for this preprint (which was not certified by peer review) is the author/funder, who has granted bioRxiv a license to display the preprint in perpetuity. It is made available under aCC-BY 4.0 International license.

# Supplementary Figure 2. Quantification of size of distal area and the intensity of distal ciliary proteins along the MCC cilia

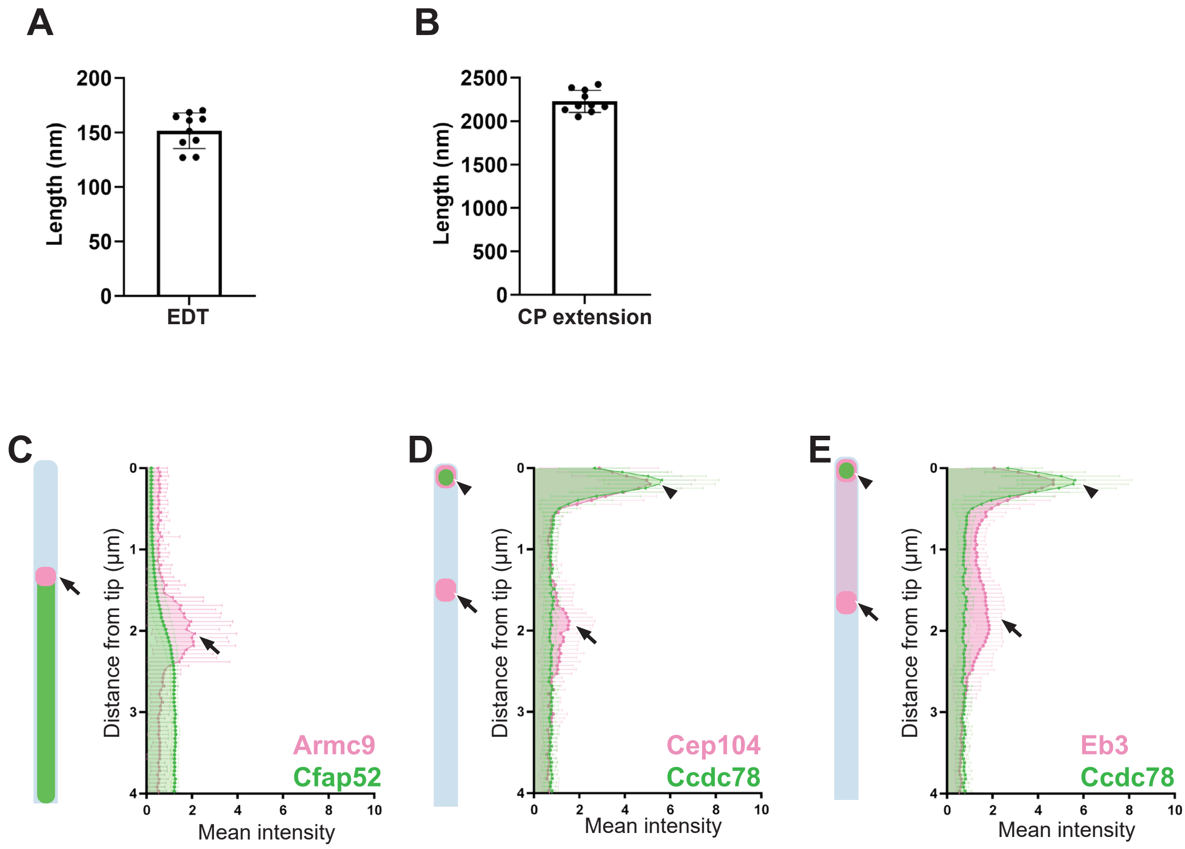

bioRxiv preprint doi: https://doi.org/10.1101/2025.02.19.639145; this version posted June 25, 2025. The copyright holder for this preprint (which was not certified by peer review) is the author/funder, who has granted bioRxiv a license to display the preprint in perpetuity. It is made available under aCC-BY 4.0 International license.

# Supplementary Figure 3. RT-PCR of Ccdc78 splicing morpholino injected embryos and effect of Ccdc78 MO on basal bodies and Eb3

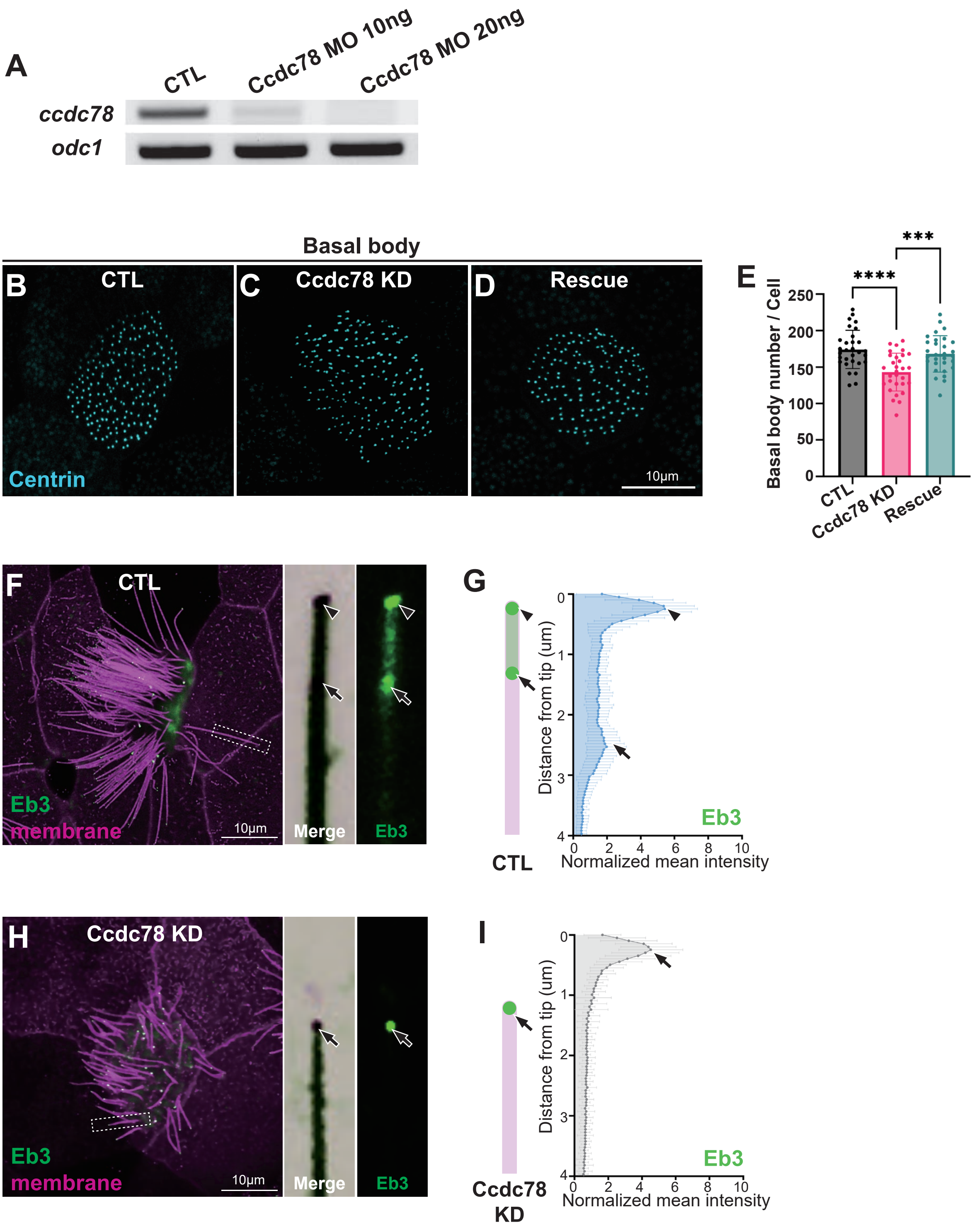

bioRxiv preprint doi: https://doi.org/10.1101/2025.02.19.639145; this version posted June 25, 2025. The copyright holder for this preprint (which was not certified by peer review) is the author/funder, who has granted bioRxiv a license to display the preprint in perpetuity. It is made available under aCC-BY 4.0 International license.

# Supplementary Figure 4. Localization of Ccdc33 in *Xenopus* MCC and Human HTEC cell

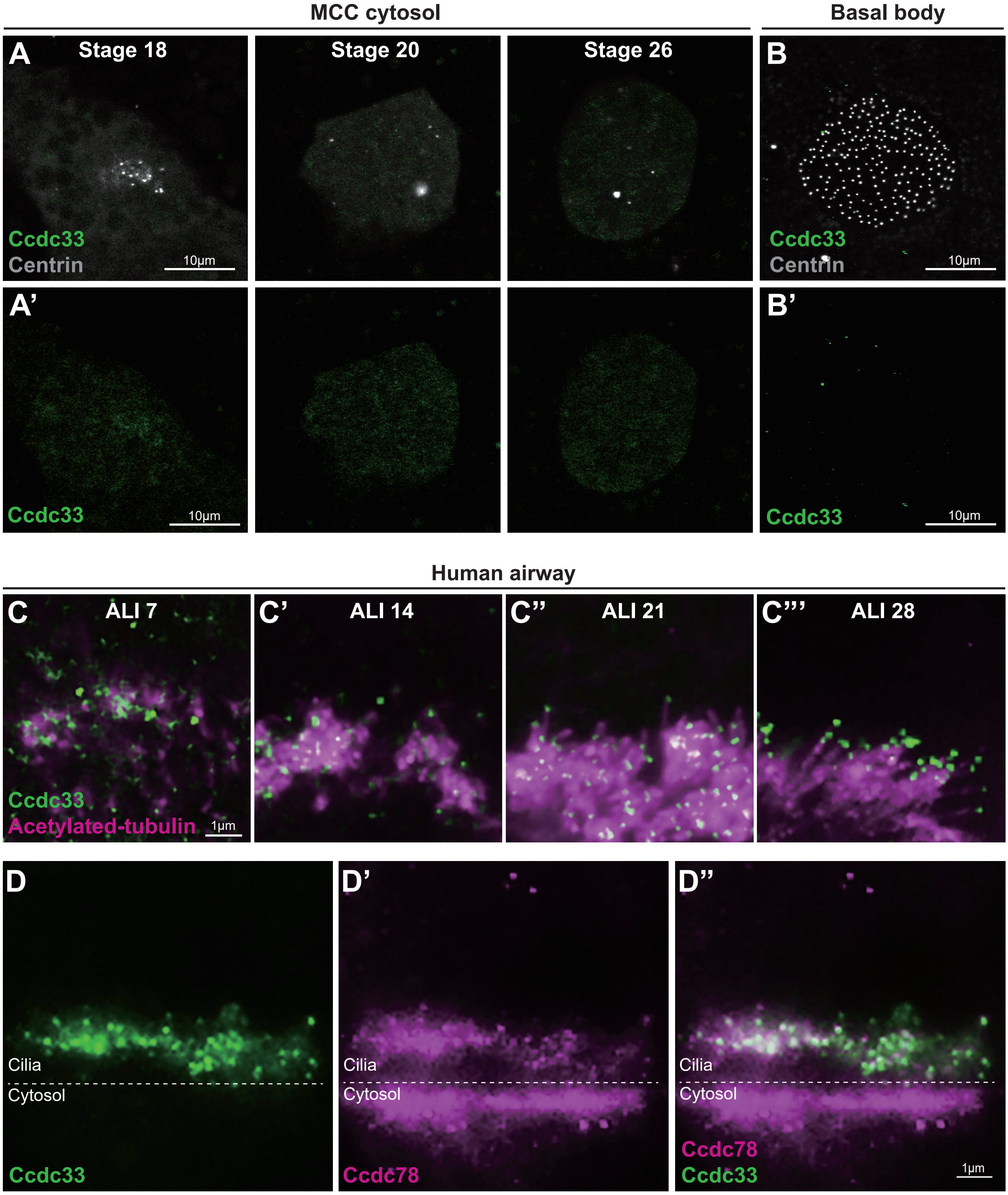

bioRxiv preprint doi: https://doi.org/10.1101/2025.02.19.639145; this version posted June 25, 2025. The copyright holder for this preprint (which was not certified by peer review) is the author/funder, who has granted bioRxiv a license to display the preprint in perpetuity. It is made available under aCC-BY 4.0 International license.

**Supplementary Figure 5. Confirmation of Ccdc33 MO with RT-PCR and total protein of Ccdc78 or Ccdc33 after knockdowns. Distribution of Eb3 after Ccdc33 knockdown.**

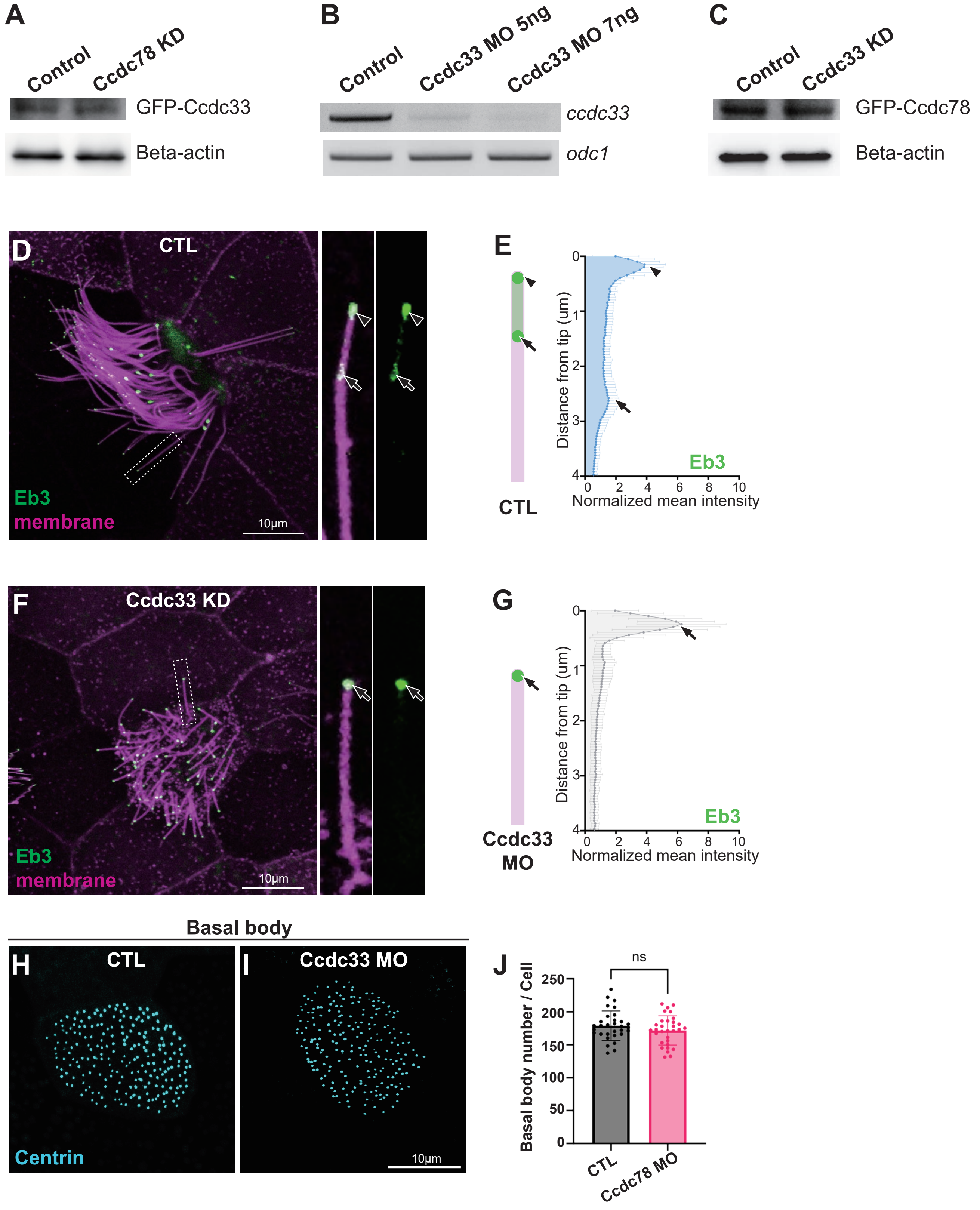

bioRxiv preprint doi: https://doi.org/10.1101/2025.02.19.639145; this version posted June 25, 2025. The copyright holder for this preprint (which was not certified by peer review) is the author/funder, who has granted bioRxiv a license to display the preprint in perpetuity. It is made available under aCC-BY 4.0 International license.

# Supplementary Figure 6. Western blots of overexpressed Myc-Ccdc78 or Myc-Ccdc33 and whole epithelium overexpression of Myc-Ccdc78 or Myc-Ccdc33

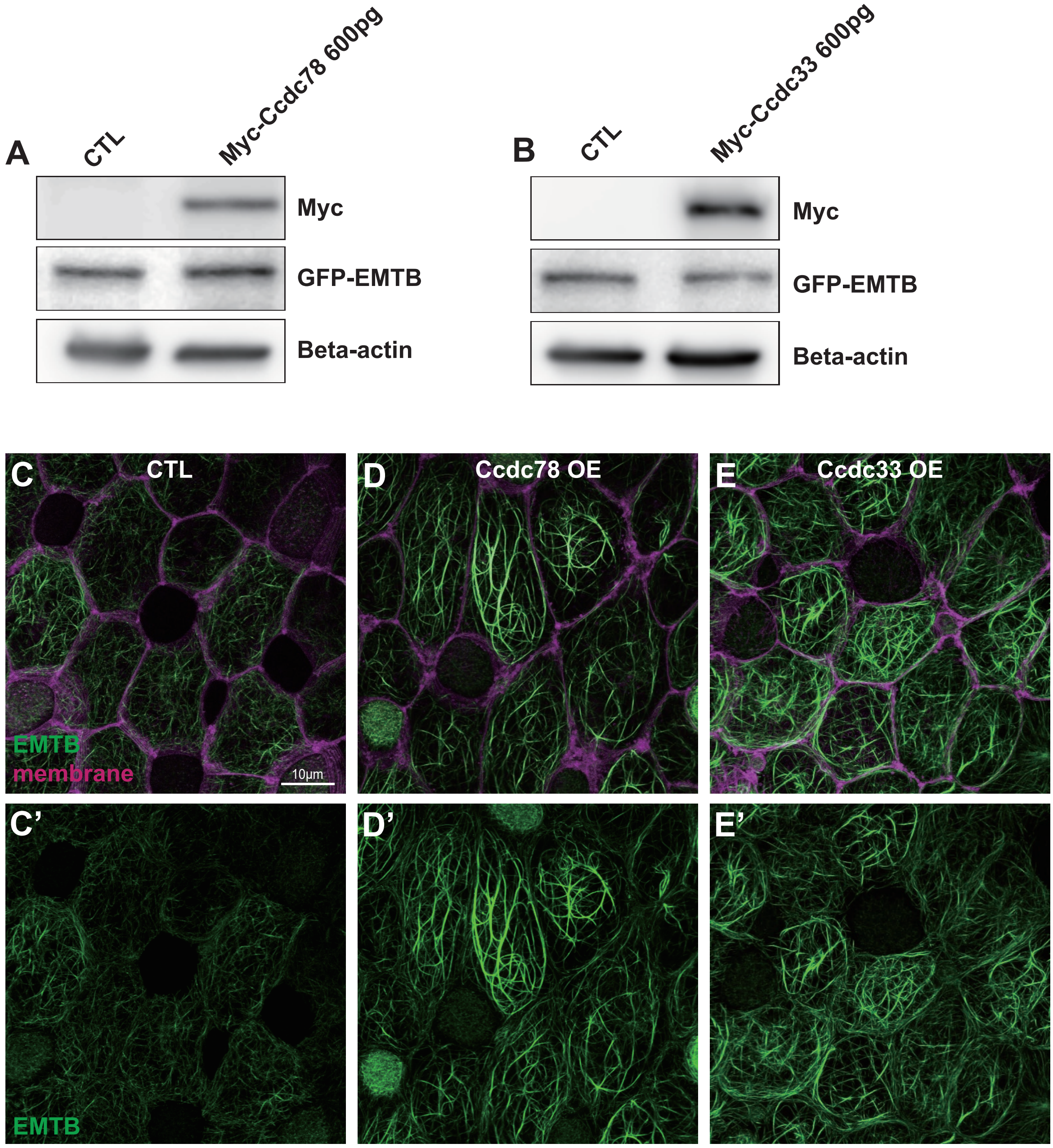

bioRxiv preprint doi: https://doi.org/10.1101/2025.02.19.639145; this version posted June 25, 2025. The copyright holder for this preprint (which was not certified by peer review) is the author/funder, who has granted bioRxiv a license to display the preprint in perpetuity. It is made available under aCC-BY 4.0 International license.

# Supplementary Figure 7. Localization of Ccdc78 and Ccdc33 in Xenopus gastrocoel roof plate 9+0 cilia

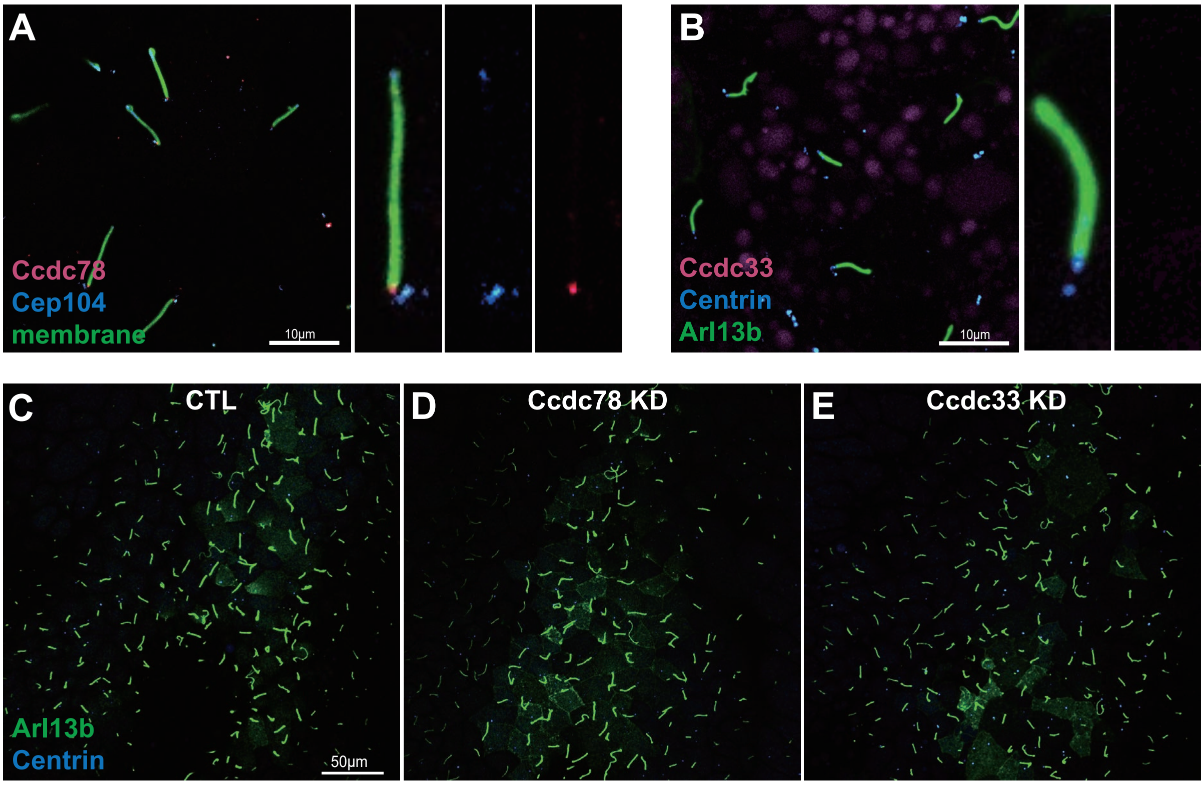

bioRxiv preprint doi: https://doi.org/10.1101/2025.02.19.639145; this version posted June 25, 2025. The copyright holder for this preprint (which was not certified by peer review) is the author/funder, who has granted bioRxiv a license to display the preprint in perpetuity. It is made available under aCC-BY 4.0 International license.

# Supplementary Figure 8. Bead flow analysis of control, Ccdc78 KD and Ccdc33 KD embryos

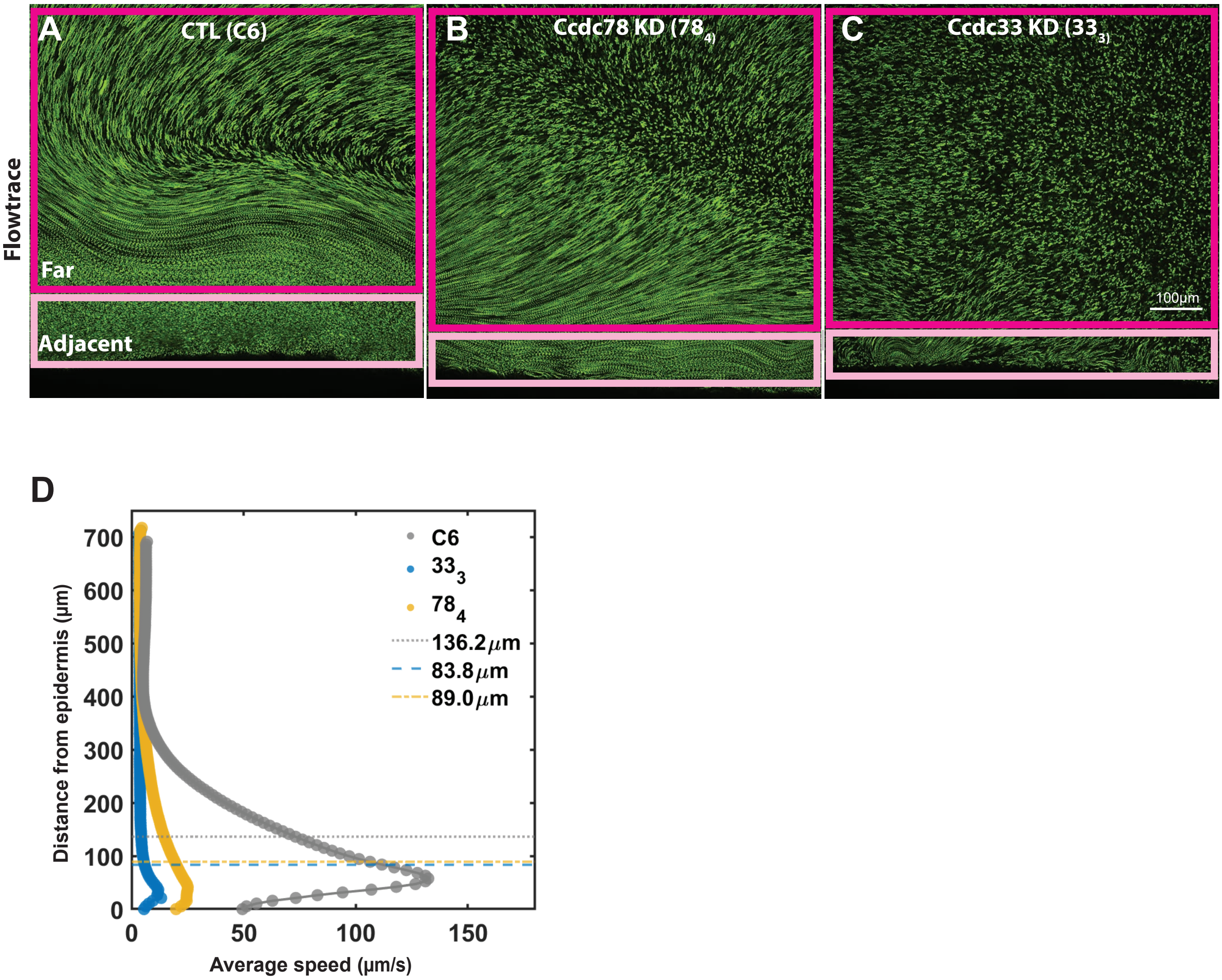

bioRxiv preprint doi: https://doi.org/10.1101/2025.02.19.639145; this version posted June 25, 2025. The copyright holder for this preprint (which was not certified by peer review) is the author/funder, who has granted bioRxiv a license to display the preprint in perpetuity. It is made available under aCC-BY 4.0 International license.

# Supplementary Figure 9. Structural difference of Ccdc78 and Ccdc33 between *Tetrahymena* and *Xenopus laevis*

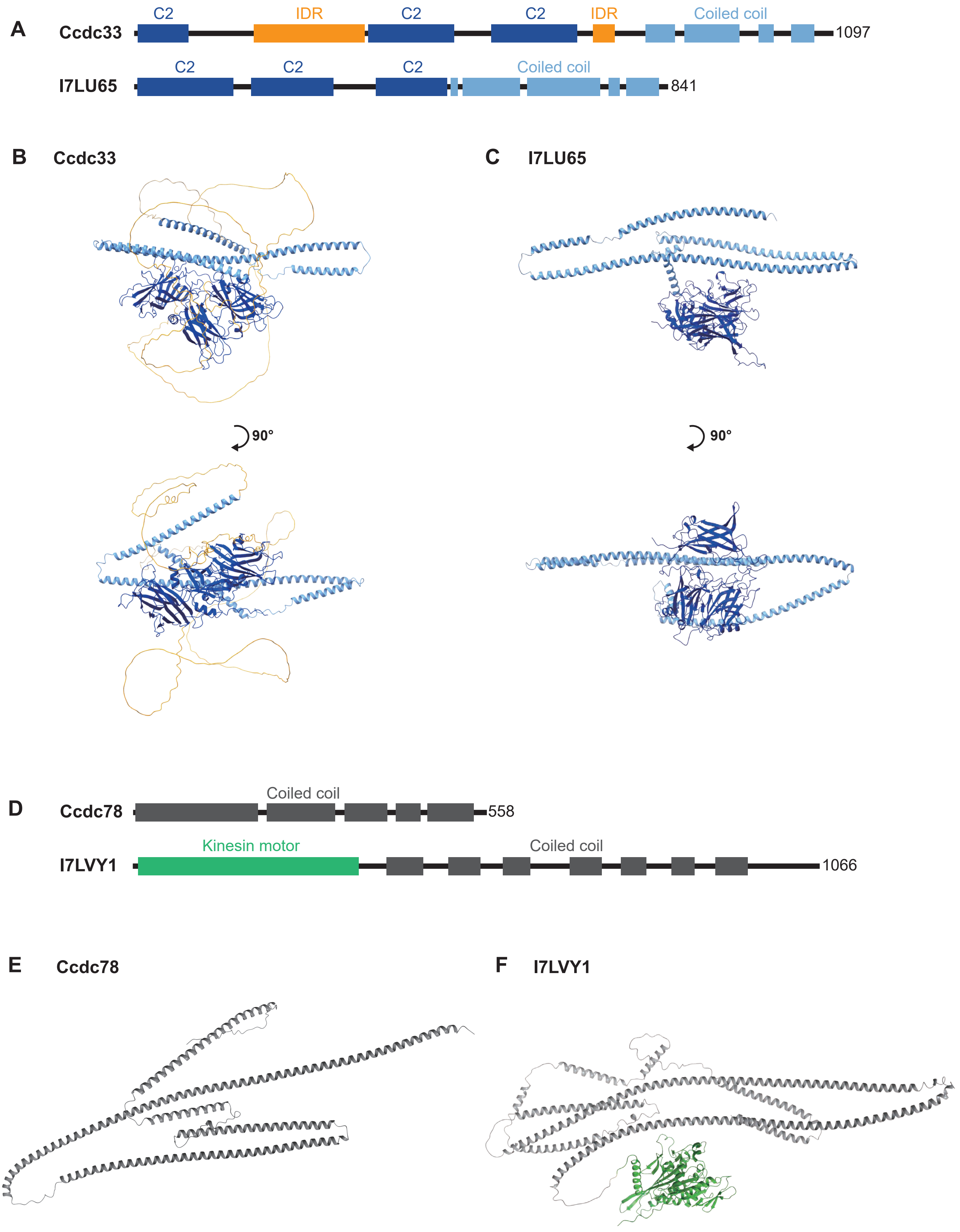

Supplement: 1 — Supplementary figure 1. Localization of Ccdc78 in MCC cytosol during ciliogenesis (A) Apical surface of Xenopus embryo epithelium of stage 18, 20, 22, and 26, showing the localization of GFP-Ccdc78 (green) with Membrane-RFP (blue). Insets show magnified views of cilia. Scale bars represent 10μm. (B) Image of Xenopus MCC cytosol expressed with GFP-Ccdc78 (green) and Centrin-BFP (gray) in stage 18, 20 and 26 embryos. Localization of GFP-Ccdc78 on the basal body of MCC is shown on the right. Scale bars represent 10μm. (C) Human trachea epithelial cells (HTEC) MCC stained with anti-acetylated tubulin (magenta) and anti-Ccdc78 (green) antibody during the time of ALI-culture day 7, 14, 21 and 28. Scale bar represents 1μm. Supplementary figure 2. Quantification of distal ciliary proteins localization along the MCC cilia (A) Quantification of the length of extreme distal tip (EDT) region in TEM images of Xenopus MCC cilia. (n=10 cilia) (B) Quantification of the length of central pair (CP) extension region in TEM images of Xenopus MCC cilia. (n=10 cilia) (C-E) Quantification of the normalized mean intensity distribution of distal proteins Armc9 with Cfap52 (C), Cep104 (D) and Eb3 (E) shown in Fig. 1I, J, K. Arrowhead and arrow pointing at the enrichment of proteins respectively. (n=40 cilia) Supplementary figure 3. RT-PCR of Ccdc78 splice-blocking morpholino-injected embryos and quantification of distal protein distributions (A) Gel image of RT-PCR showing ccdc78 and odc1 mRNA levels in control embryos and embryos injected with 10 ng or 20ng of Ccdc78 MO. (B-E)) Image of basal bodies expressed with Centrin-BFP (cyan) in control (B), Ccdc78 KD (C) and rescue (D) embryos. Scale bar represents 10 μm. (E) Quantification of the number of basal bodies per cell is shown on the right. ****P<0.0001, **P<0.01. (n=31 cells) (Ordinary one-way ANOVA). (F-I) Confocal image of membrane-RFP with GFP-Eb3 (arrowhead and arrow) in control (F-G) and Ccdc78 KD (H-I) MCC cilia with magnified view [file NIHPP2025.02.19.639145V2-supplement-1.pdf]
