# Supplementary material for: Molecular organization of the distal tip of vertebrate motile cilia
Source: bioRxiv. 2025 Feb 19:2025.02.19.639145. Preprint. [Version 1] doi: 10.1101/2025.02.19.639145 (PMC11870508; doi:10.1101/2025.02.19.639145)

Supplementary Figure 1. Localization of Ccdc78 *Xenopus* MCC and human trachea epithelial cells along the developmental stages

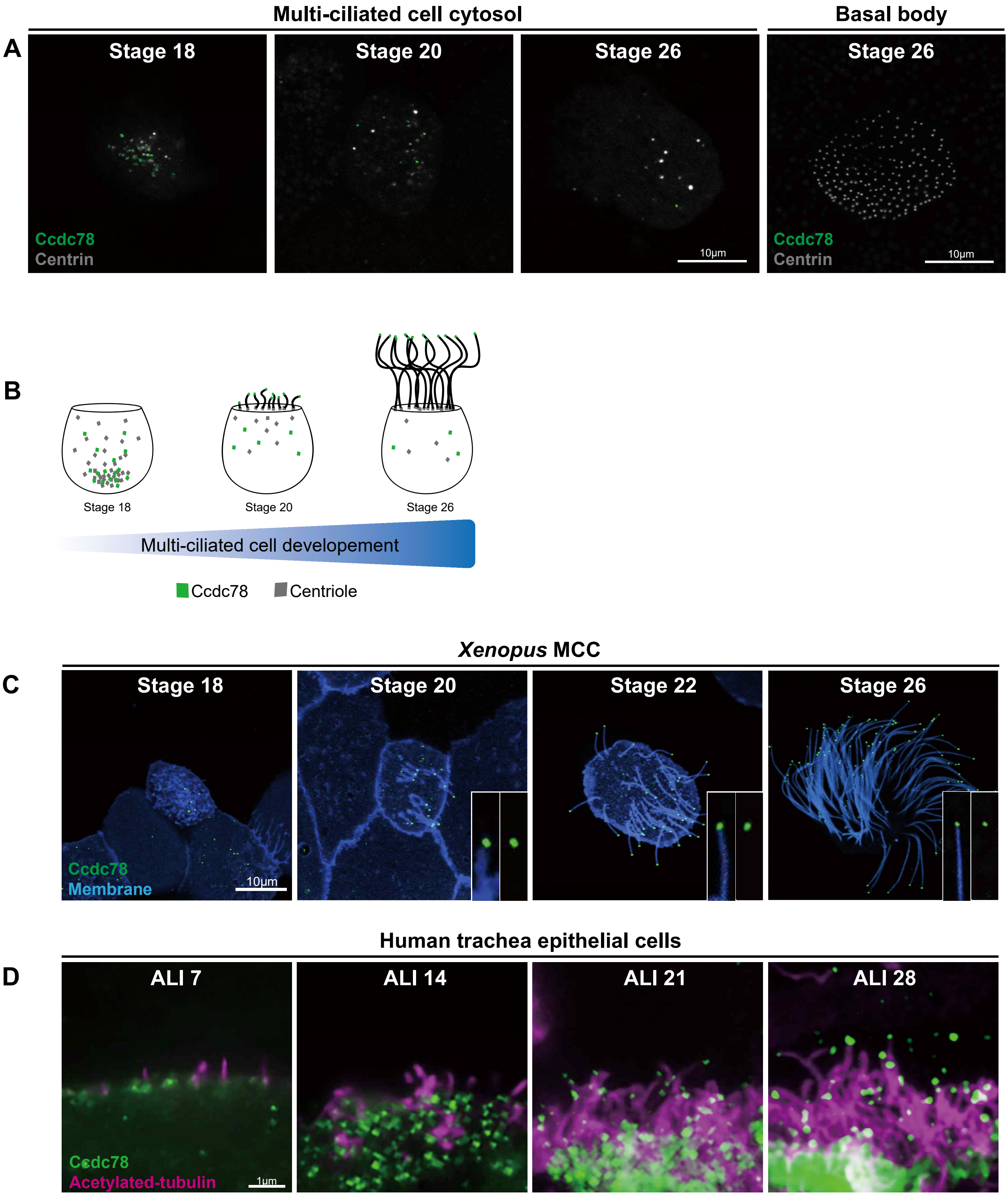

bioRxiv preprint doi: https://doi.org/10.1101/2025.02.19.639145; this version posted February 19, 2025. The copyright holder for this preprint (which was not certified by peer review) is the author/funder, who has granted bioRxiv a license to display the preprint in perpetuity. It is made available under aCC-BY 4.0 International license.

Supplementary Figure 2. RT-PCR of Ccdc78 splicing morpholino injected embryos and effect of Ccdc78 MO on basal bodies and distal ciliary proteins

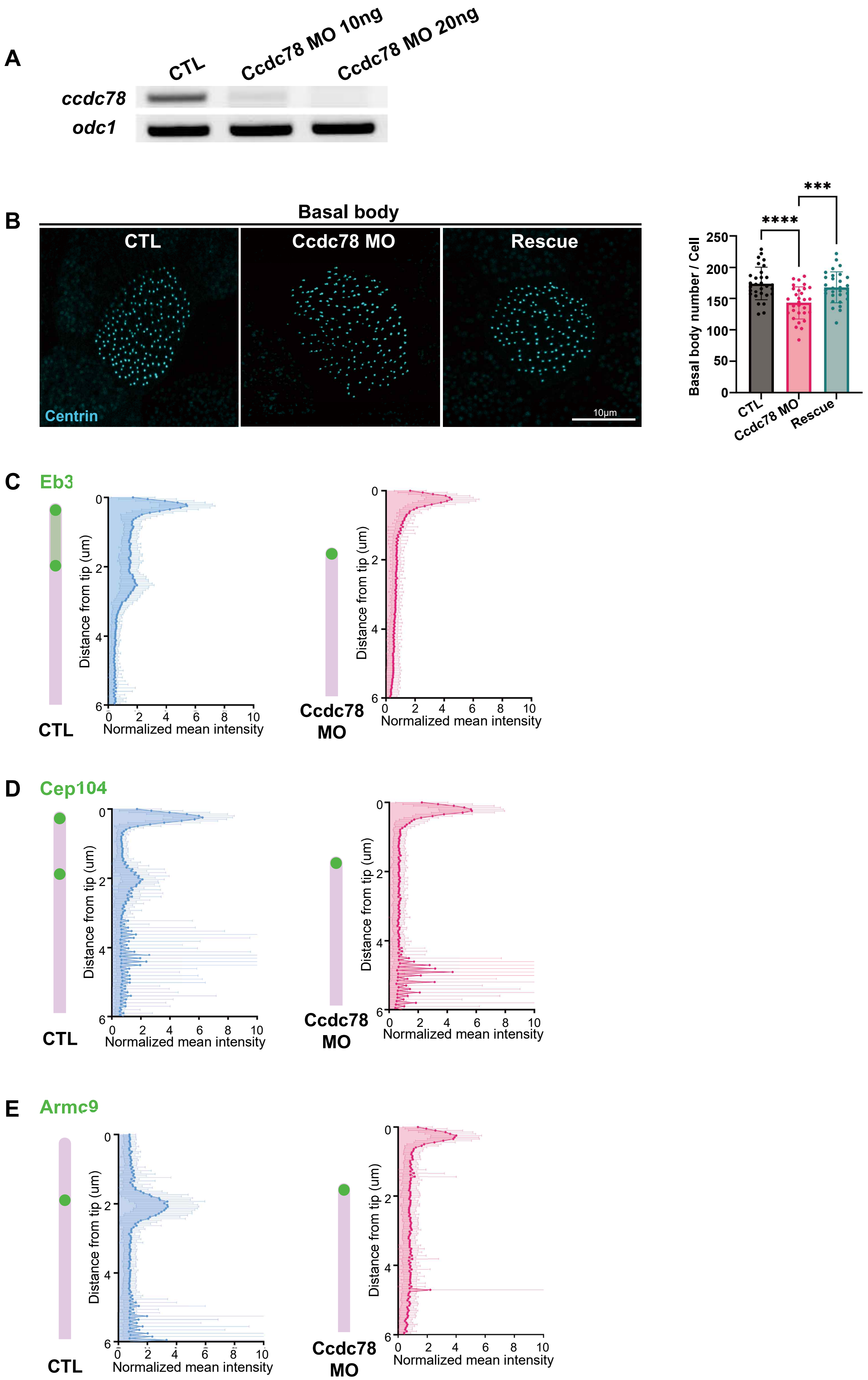

bioRxiv preprint doi: https://doi.org/10.1101/2025.02.19.639145; this version posted February 19, 2025. The copyright holder for this preprint (which was not certified by peer review) is the author/funder, who has granted bioRxiv a license to display the preprint in perpetuity. It is made available under aCC-BY 4.0 International license.

Supplementary Figure 3. AP-MS of *Xenopus* animal caps and localization of Tsga10 in *Xenopus* MCC

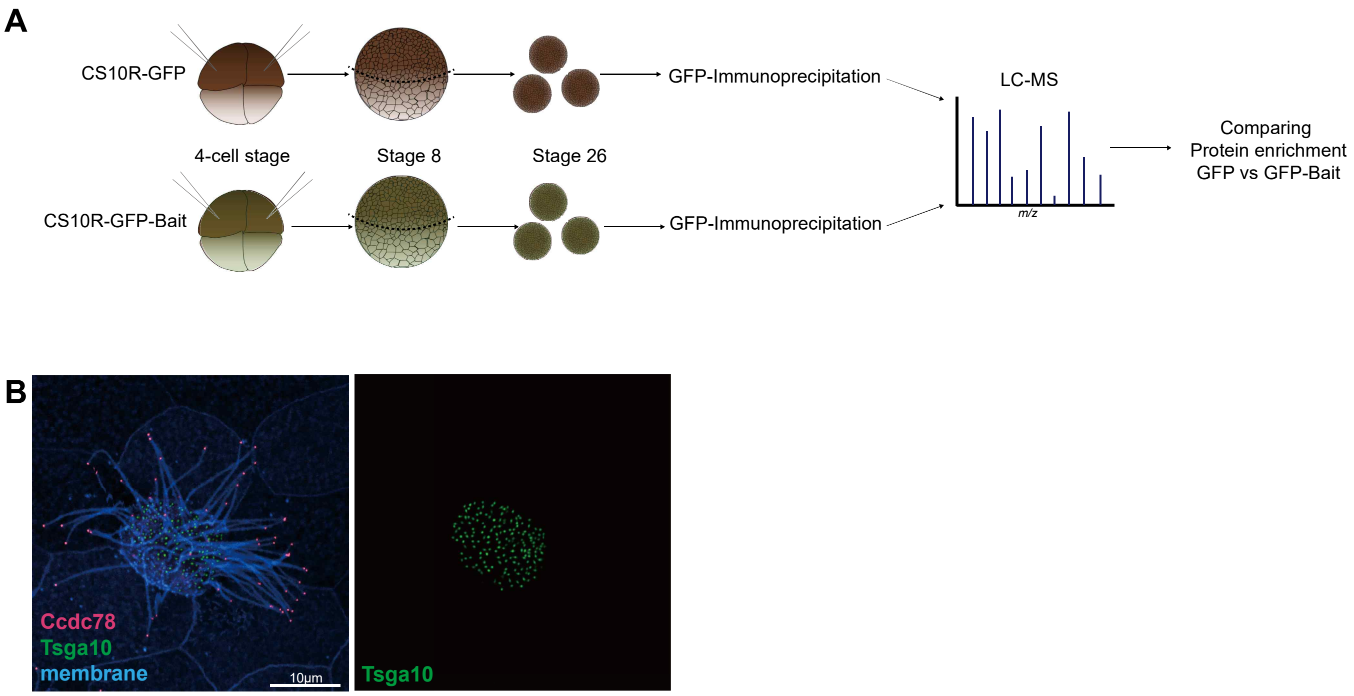

bioRxiv preprint doi: https://doi.org/10.1101/2025.02.19.639145; this version posted February 19, 2025. The copyright holder for this preprint (which was not certified by peer review) is the author/funder, who has granted bioRxiv a license to display the preprint in perpetuity. It is made available under aCC-BY 4.0 International license.

Supplementary Figure 4. Localization of Ccdc33 in *Xenopus* MCC and Human HTEC cell

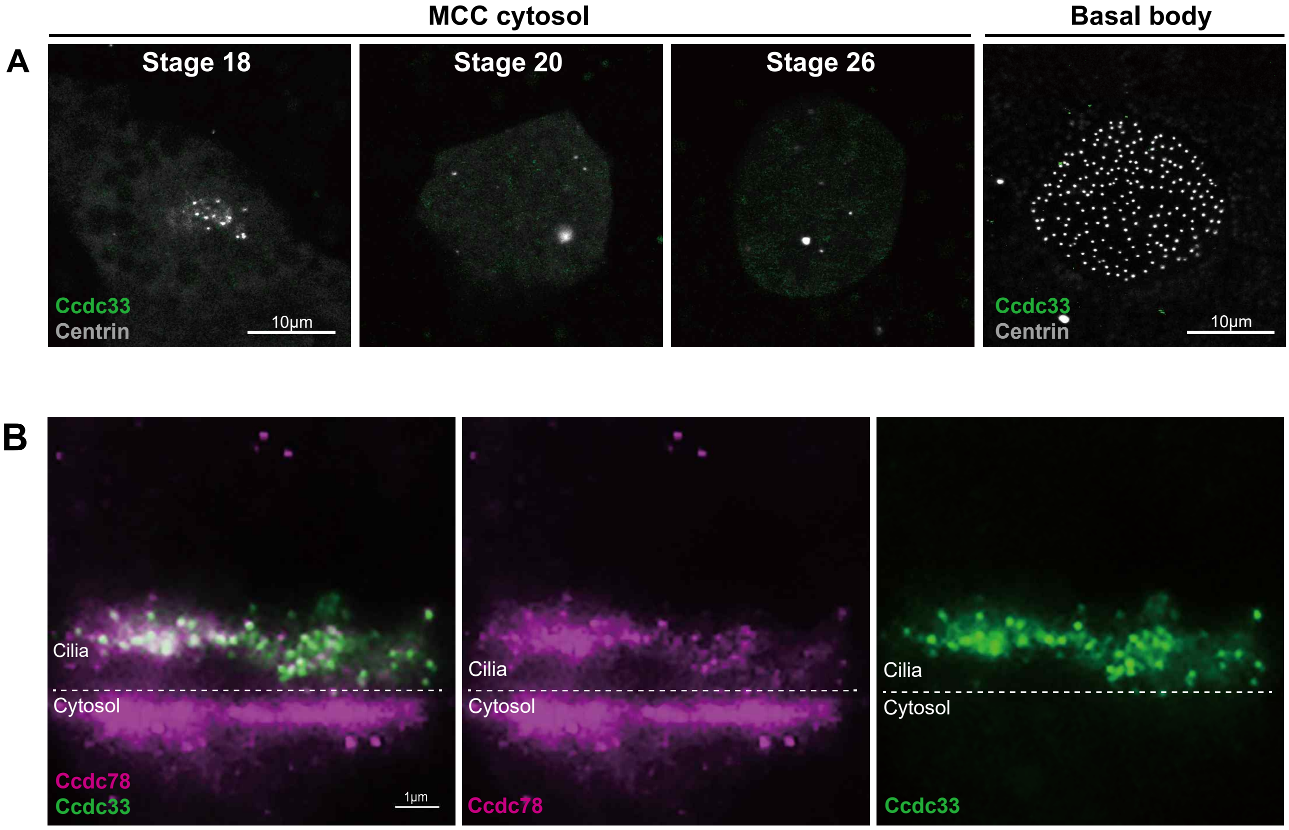

bioRxiv preprint doi: https://doi.org/10.1101/2025.02.19.639145; this version posted February 19, 2025. The copyright holder for this preprint (which was not certified by peer review) is the author/funder, who has granted bioRxiv a license to display the preprint in perpetuity. It is made available under aCC-BY 4.0 International license.

Supplementary Figure 5. RT-PCR of Ccdc33 splice-blocking morpholino and distribution of Ccdc78 and Ccdc33 intensity along the cilia upon Ccdc33 and Ccdc78 morpholino injection

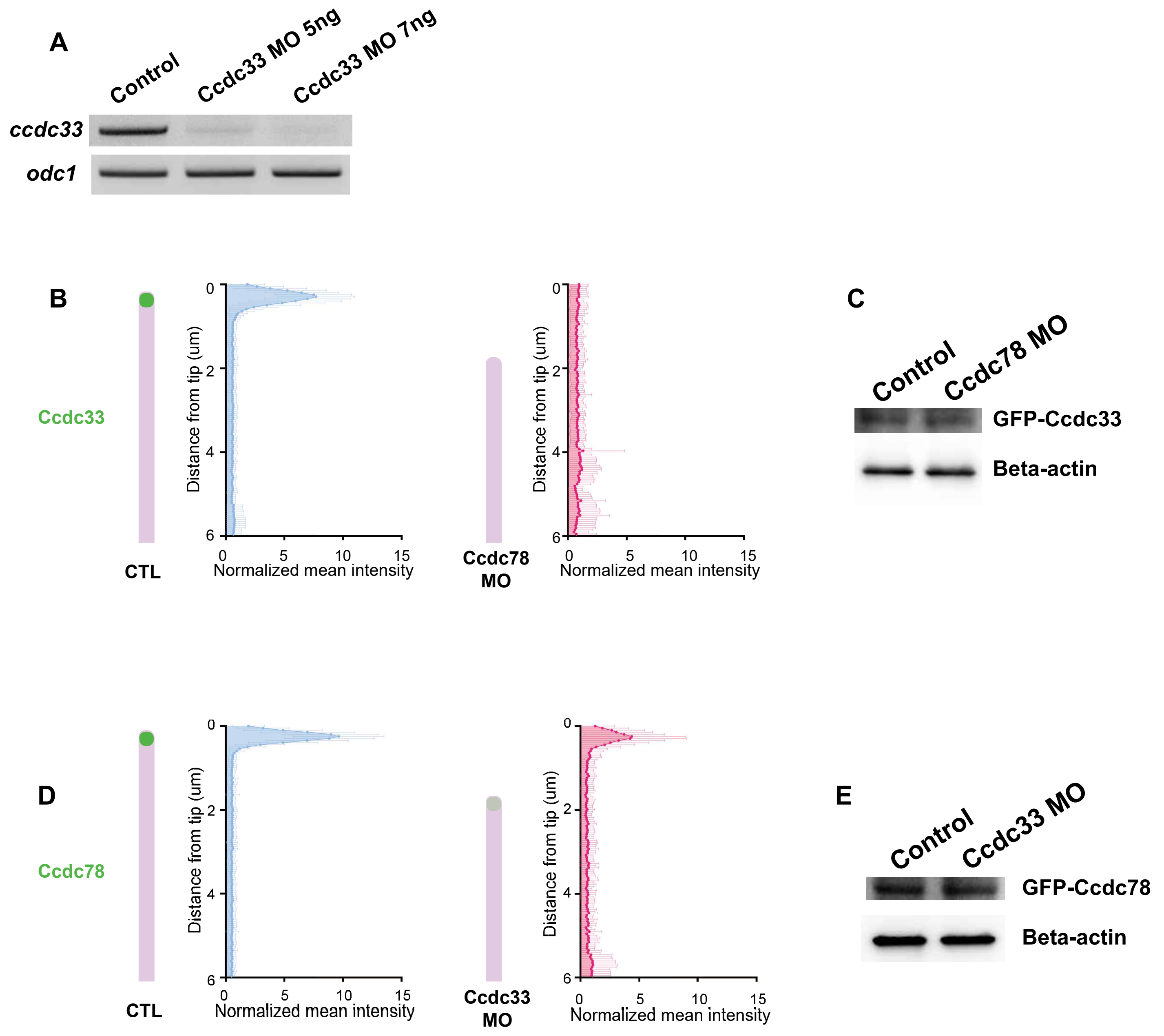

bioRxiv preprint doi: https://doi.org/10.1101/2025.02.19.639145; this version posted February 19, 2025. The copyright holder for this preprint (which was not certified by peer review) is the author/funder, who has granted bioRxiv a license to display the preprint in perpetuity. It is made available under aCC-BY 4.0 International license.

Supplementary Figure 6. Knockdown effect of Ccdc33 on basal bodies and distal tip proteins in MCC

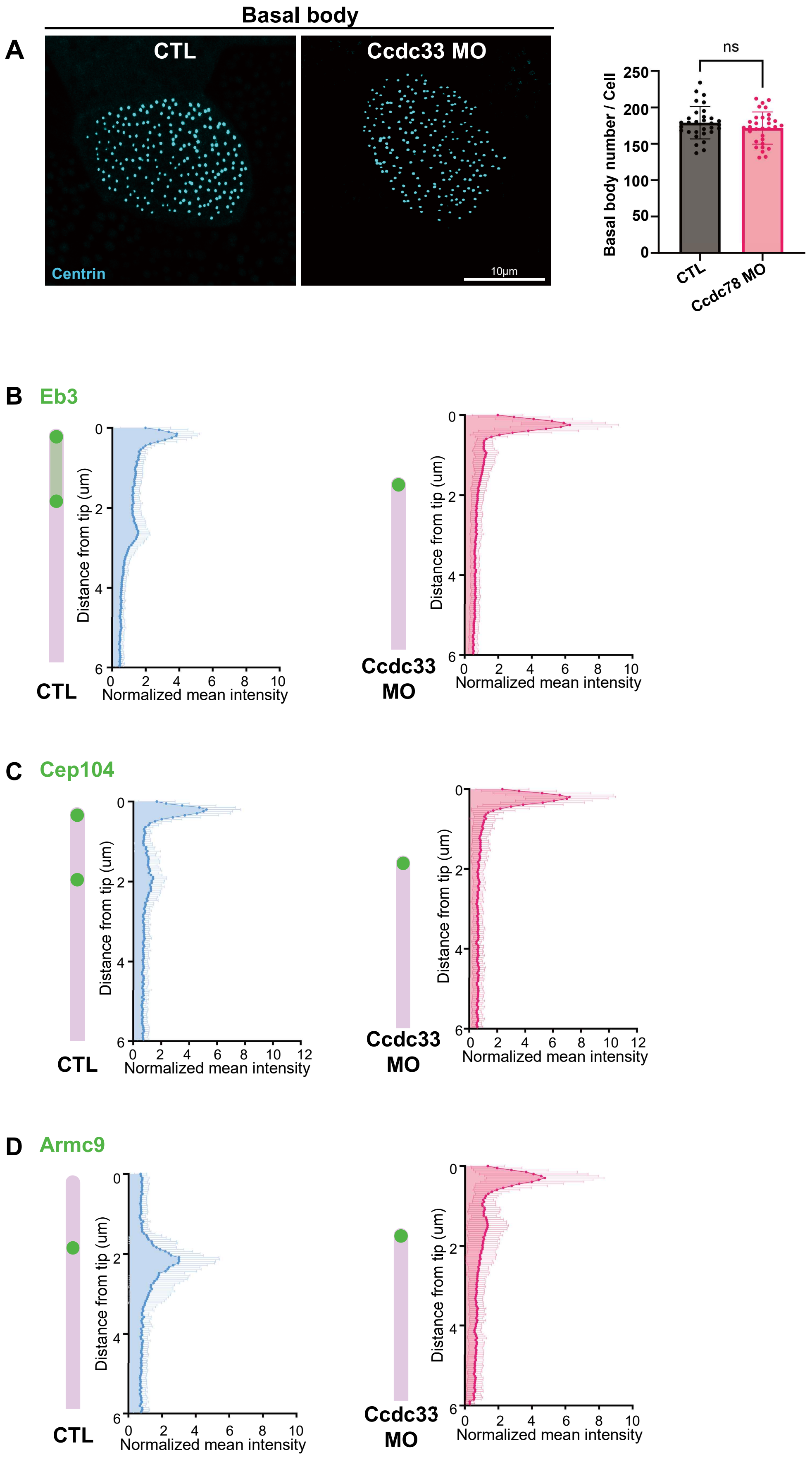

bioRxiv preprint doi: https://doi.org/10.1101/2025.02.19.639145; this version posted February 19, 2025. The copyright holder for this preprint (which was not certified by peer review) is the author/funder, who has granted bioRxiv a license to display the preprint in perpetuity. It is made available under a CC-BY 4.0 International license.

Supplementary Figure 7. Ccdc78 and Ccdc33 specifically affect 9+2 cilia in MCC

A Tailbud stage 26

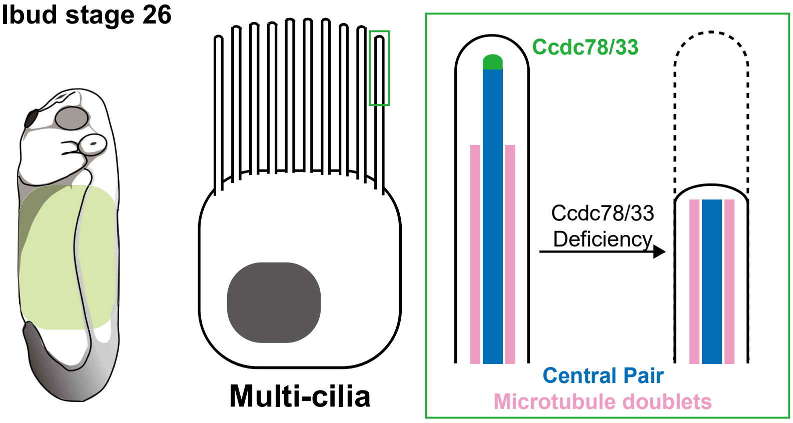

B Neurula stage 15

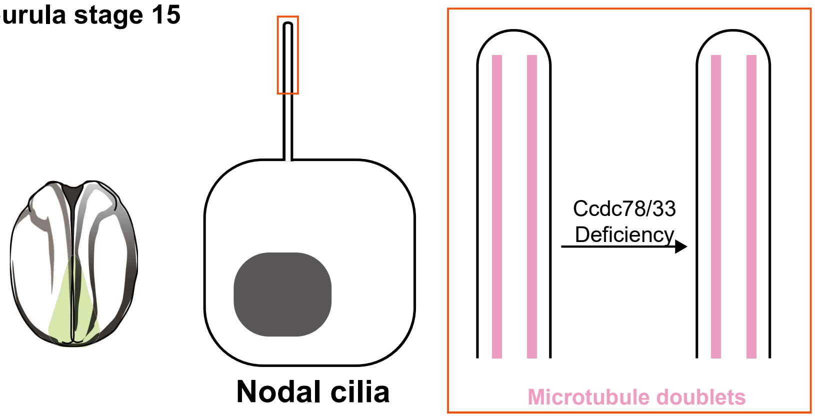

bioRxiv preprint doi: https://doi.org/10.1101/2025.02.19.639145; this version posted February 19, 2025. The copyright holder for this preprint (which was not certified by peer review) is the author/funder, who has granted bioRxiv a license to display the preprint in perpetuity. It is made available under aCC-BY 4.0 International license.

Supplementary Figure 8: Mosaics on *Xenopus* embryo epithelium and Western blots of Myc-Ccdc78 and Myc-Ccdc33

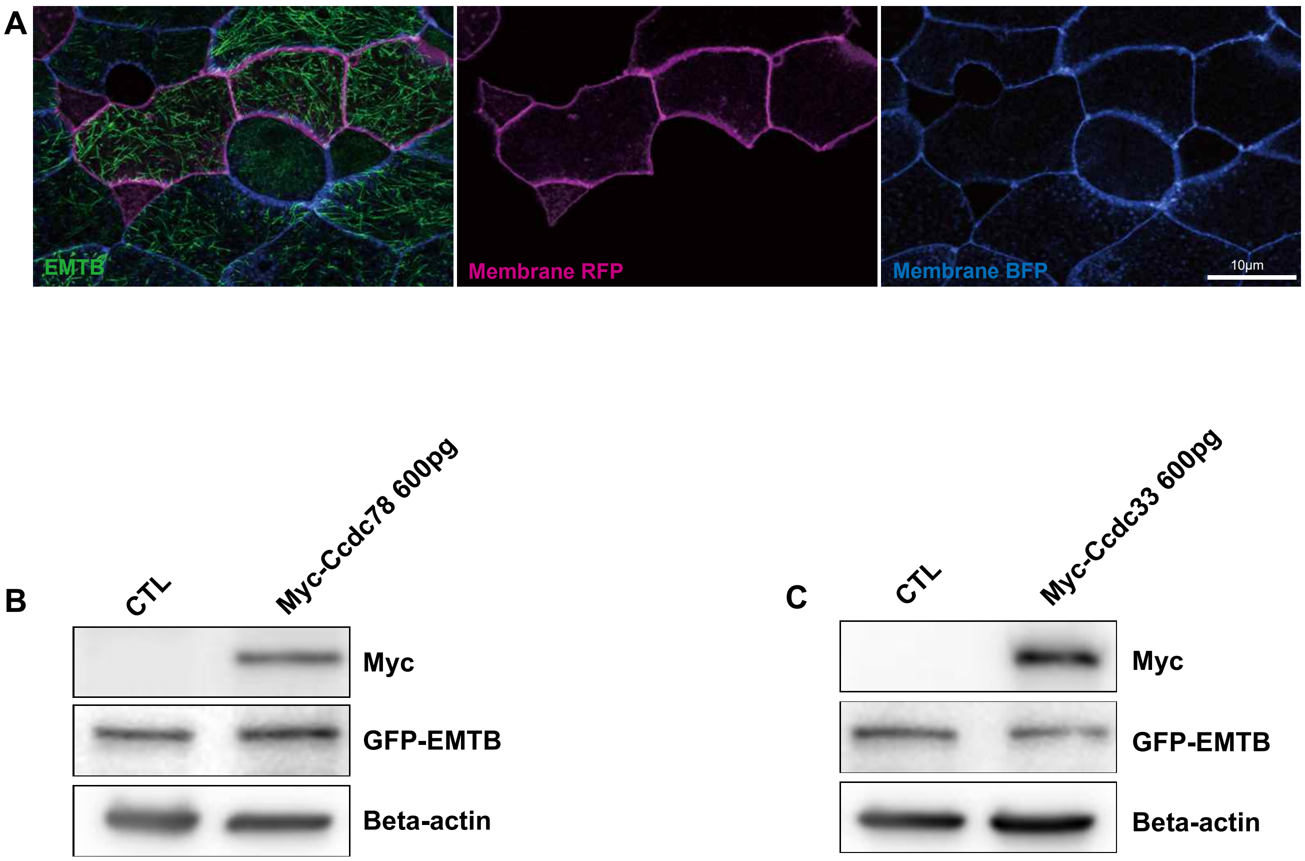

bioRxiv preprint doi: https://doi.org/10.1101/2025.02.19.639145; this version posted February 19, 2025. The copyright holder for this preprint (which was not certified by peer review) is the author/funder, who has granted bioRxiv a license to display the preprint in perpetuity. It is made available under aCC-BY 4.0 International license.

Supplementary Figure 9. Structural difference of Ccdc78 and Ccdc33 between *Tetrahymena* and *Xenopus laevis*

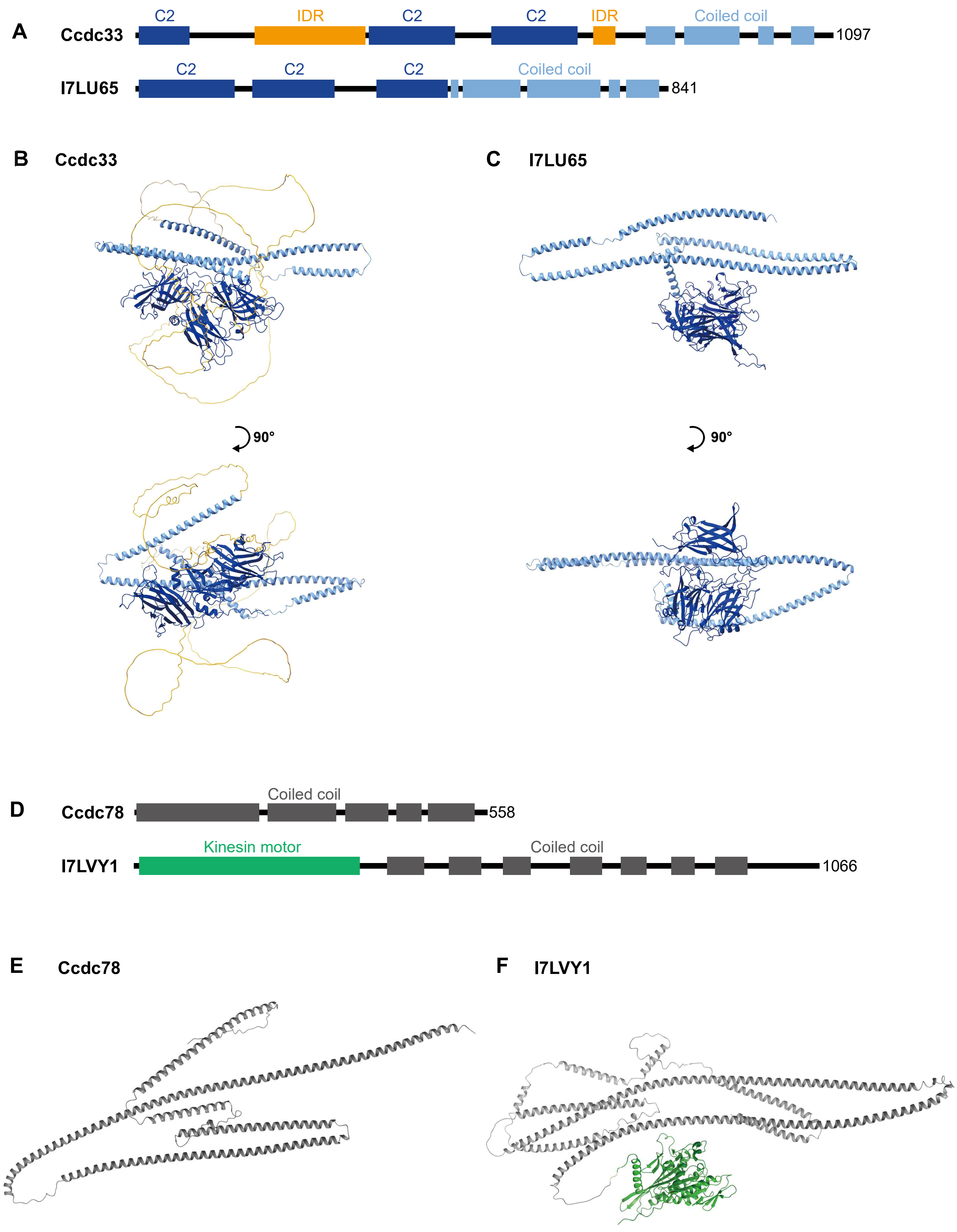

Supplement: Supplementary file 1 [file NIHPP2025.02.19.639145V1-supplement-1.pdf]
